# Cross-Domain Chest Radiograph Report Generation Under Dataset Shift: Bidirectional Audit Study

**DOI:** 10.2196/95888

**Published:** 2026-10-21

**Authors:** Yishan Sun, Jun Jiang, Yu Chen

**Affiliations:** 1 School of Electronics and Information Engineering, Sichuan University, Sichuan University Wangjiang Campus, 24 South Section 1, First Ring Road, Chengdu, Sichuan, 610065, China, 86 1-808-110-0530; 2 School of Communication and Information Engineering, Shanghai University, Shanghai, China

**Keywords:** chest radiograph report generation, cross-domain generalization, dataset shift, audit-oriented evaluation, report recurrence, clinical omission, unsupported findings, case-specific informativeness, medical vision-language models

## Abstract

**Background:**

Cross-domain chest radiograph report generation may produce formally valid reports that are nevertheless repetitive, weakly case-specific, or missing clinically relevant findings. These failures can be missed when evaluation relies on a single language-overlap or clinical-label score.

**Objective:**

This study aimed to evaluate cross-domain report generation with a bidirectional layered audit, characterize direction-specific and end point–specific failure profiles under a composite training-side control and 3 decoding policies, and explore whether selected audit dimensions remained informative for a contemporary medical vision language model.

**Methods:**

A fixed R2Gen-style system was evaluated bidirectionally on 575 IU X-ray and 401 MIMIC-CXR (Medical Information Mart for Intensive Care Chest X-Ray) target records. The audit included surface validity, complete-report recurrence and concentration, a predefined prefix end point, lexical diversity, official Stanford CheXpert/NegBio labels, paired cluster-bootstrap inference, and blinded review by 2 medical trainees. The primary human audit included 210 reports from 70 randomly sampled cases, with blinded adjudication of unresolved primary end point disagreements. A 60-report targeted panel was used for failure-mode analysis. Separately, a fixed MedGemma 1.5 4B configuration generated reports for all 976 records.

**Results:**

In MIMIC-CXR–to–IU X-ray transfer, out of 575 reports, ON (protocol-defined training-side control enabled)+B4 (beam search with a beam size of 4) reduced the predefined recurrent prefix from 575 (100%) to 8 (1.4%; Holm-adjusted *P*=.002). However, 569 (99.0%) ON+B4 outputs still belonged to exact duplicate groups. In IU X-ray–to–MIMIC-CXR transfer, the 14-category CheXpert/NegBio micro-*F*_1_ was higher for ON+B4 than for OFF (protocol-defined training-side control disabled)+B4 (0.1687 vs 0.0744; Holm-adjusted *P*=.01). This apparent advantage was not broad-based: after cardiomegaly was excluded in a post hoc descriptive sensitivity analysis, the difference changed from +0.0944 to −0.0610. Text-level tracing showed that the original contrast was mainly driven by a labeler response to recurrent, semantically normal heart-size wording. Across the primary human-audit cells, major omissions occurred in 12 to 15 out of 35 reports, and major unsupported findings occurred in 10 to 16 of 35 reports, with no consistent configuration ordering observed. The exploratory MedGemma micro-*F*_1_ was 0.4722 on IU X-ray and 0.4340 on MIMIC-CXR, but 381 out of 575 (66.3%) IU outputs belonged to exact duplicate groups.

**Conclusions:**

No configuration performed consistently well across end points, and no single metric captured all forms of cross-domain degradation. The MedGemma stress test suggested that recurrence and label-traceability checks remain useful across architectures, although it was not a matched performance comparison. Cross-domain report generators should undergo bidirectional evaluation, final-text verification, and image-grounded human review before external validation or clinical deployment.

## Introduction

### Background and Evaluation Problem

Chest radiograph report generation combines image understanding and clinical language generation. Although recent multimodal systems perform well on in-domain automatic metrics [1-5], rule-based clinical labeling with CheXpert and NegBio has become a common evaluation component [6,7]. Meanwhile, retrieval, progressive-generation, relation-extraction, and hallucination-reduction methods have expanded the field [8-12]; however, cross-domain behavior remains less understood.

Cross-domain deployment is, nevertheless, realistic [13,14]. Institutional reporting style, disease prevalence, acquisition patterns, language, and annotation practices can shift model behavior, leaving fluent reports that are generic, repetitive, and weakly case specific.

No single metric adequately captures this degradation. Language-overlap measures may miss clinically important omissions or template collapse [3,5,15], while label-based agreement remains a proxy rather than a form of report-level factual verification. Therefore, plausible form need not imply useful case-specific content [15-17].

Cross-domain report generation should, therefore, be treated as both a generation and an evaluation problem. A layered audit can distinguish structural failure, recurrent or generic output, weak proxy alignment, and loss of clinically useful case-specific information.

### Recent Work and Remaining Gap

Recent work has extended evaluation beyond language overlap through structured-report benchmarks, clinical concept extraction, error analysis, statistical testing, reader studies, and radiologist-alignment analyses [10,15,18-21]. Model-development studies have also introduced segmentation-enhanced, abnormality-guided, and disease-aware architectures [22-25]. A more distant line of medical imaging research has examined segmentation and classification for magnetic resonance imaging tumor analysis; this image-level detection setting is not directly comparable with cross-domain radiology report generation [26].

These studies address complementary problems, but few combine symmetric source-target reversal, cluster-aware inference, final-text traceability, and independent human review in one evaluation.

### Study Objectives and Contributions

This study examined bidirectional cross-domain transfer between MIMIC-CXR (Medical Information Mart for Intensive Care Chest X-Ray) and IU X-ray as a structured evaluation problem. It addressed four questions: (1) whether a protocol-defined training-side control altered severe cross-domain failure relative to an uncontrolled reference; (2) how ON (protocol-defined training-side control enabled)+B4 (beam search with a beam size of 4), ON+G1 (greedy decoding), and ON+K50 (top-k sampling with k=50) differed in surface validity, report recurrence and concentration, protocol-defined template-prefix behavior, lexical diversity, and proxy clinical alignment; (3) whether blinded human review identified clinically relevant omissions, unsupported findings, or loss of case specificity not captured by automated measures; and, exploratorily, (4) whether selected automated audit dimensions remained informative when applied to a contemporary medical vision-language model under one fixed configuration. Figure 1 summarizes the study design.

Together, the design separates corpus-level degeneration, labeler behavior, and case-level clinical error across both transfer directions and an exploratory MedGemma stress test.

**Figure 1.**
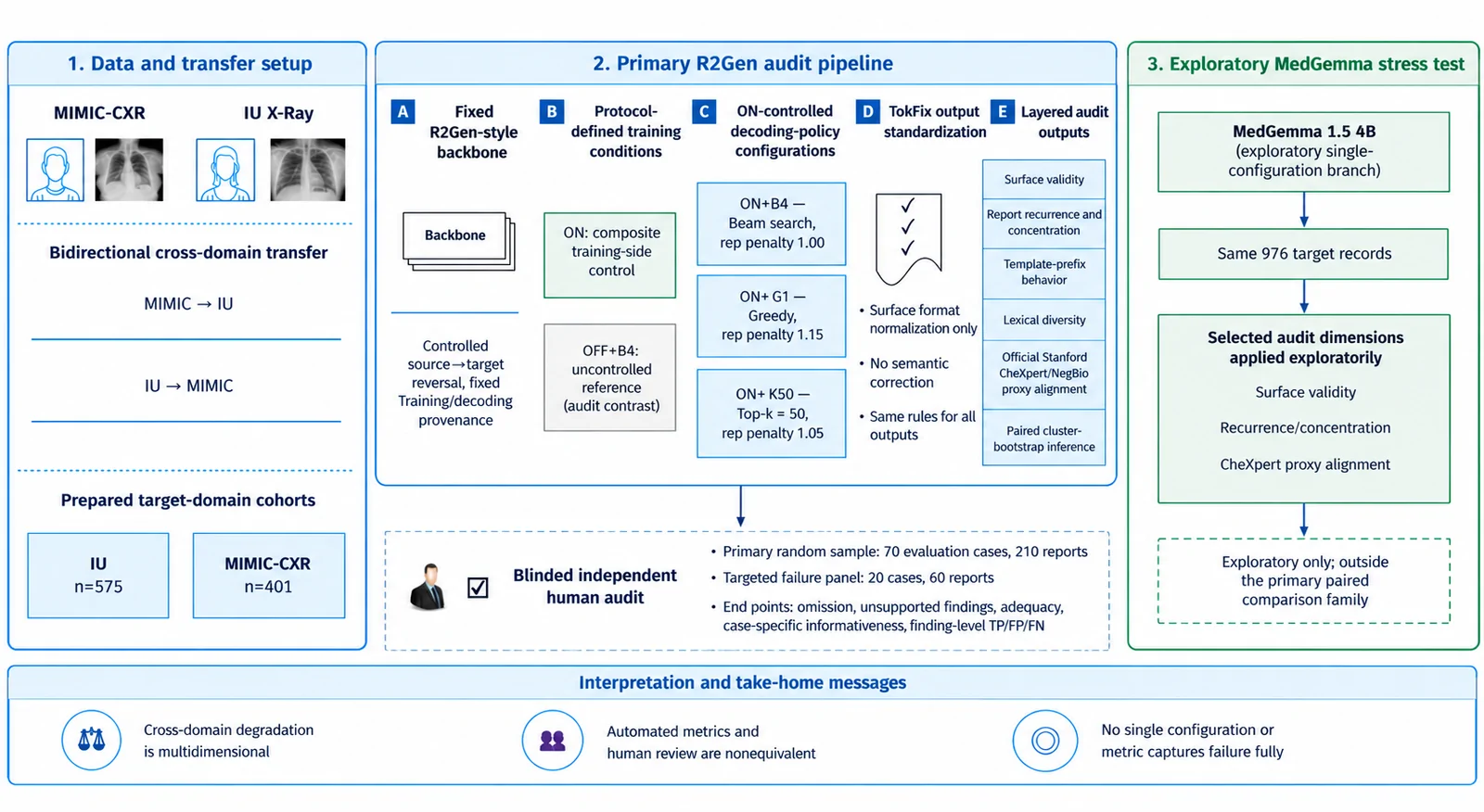
Overall study design and layered audit framework for cross-domain chest radiograph report generation. FN: false negative; FP: false positive; MIMIC-CXR: Medical Information Mart for Intensive Care Chest X-Ray; OFF: protocol-defined training-side control disabled; ON: protocol-defined training-side control enabled; TP: true positive.

## Methods

### Study Design and Analytical Framework

The layered evaluation compared the composite ON protocol with OFF (protocol-defined training-side control disabled)+B4, ON+B4, ON+G1, and ON+K50 across automated end points and used blinded review to identify clinically relevant errors. MedGemma was assessed separately under a fixed exploratory configuration.

Automated measures characterized full-cohort validity, recurrence, concentration, lexical diversity, and labeler-derived alignment; human review assessed omission, unsupported findings, adequacy, informativeness, and finding-level agreement.

ON/OFF evaluated the composite training protocol, whereas the ON-policy comparisons described residual decoding trade-offs. MedGemma was excluded from the paired comparisons and the Holm-corrected hypothesis family.

### Datasets and Evaluation Cohort Construction

The cohorts were constructed prior to training and evaluation. Figure S1 and Table S22 in Multimedia Appendix 1 present the fully reconstructed preparation flow, selection rules, and integrity checks.

For IU X-ray, prepare_iuxray.py produced 3826 eligible entries, which were split at the IU unique ID level with seed 42. The test set contained 575 entries with no unique ID overlap across partitions. Because reliable patient identifiers were unavailable, patient-level disjointness could not be confirmed; any residual patient overlap would likely make cross-domain performance appear better than it actually is and therefore underestimate transfer degradation.

For MIMIC-CXR, 5159 official-test rows were scanned in fixed metadata order. Restricting to posteroanterior, anteroposterior, or anteroposterior supine views retained 3403 rows; 29 reports were excluded because the cleaned impression+findings text contained fewer than 32 characters, leaving 3374 eligible image-level entries. The predetermined cap retained the first 401 (11.9%) entries, representing 347 studies. There was no random sampling, seed, output-based selection, or CheXpert-label filtering.

All MIMIC-CXR inputs were frontal radiographs. The IU preparation selected the first frontal projection when available and otherwise the first available projection, yielding 553 frontal and 22 lateral fallback inputs. IU references combined the findings and impression; MIMIC-CXR references used the preferred findings and impression sections. Because some reference reports summarized multiview examinations, a single model input could omit information visible only on another projection.

No configuration-specific attrition occurred after the test partitions were fixed: all 575 IU and 401 MIMIC-CXR entries had images, references, complete outputs for the 4 configurations, and successful case-reference matching.

### Generation Settings and Output Standardization

A fixed R2Gen-style backbone was used [4] to audit controlled bidirectional transfer rather than to establish state-of-the-art performance. MIMIC-CXR [27] and IU X-ray [28] served as the source and target domains. ON enabled a composite training-side control against repetitive generic output; OFF used the default cross-entropy path. MedGemma was evaluated separately as an exploratory stress test.

The ON condition combined nontemplate-balanced sampling, lag-1 and lag-2 recurrence penalties, early end-of-sequence (EOS) suppression, EOS-token reweighting, and a template-token probability penalty; the adaptive controller and late-EOS term were inactive. The OFF condition disabled these components. Because no component ablation was performed, ON/OFF effects were attributed only to the composite protocol.

Training-side marking used 6 frozen lowercase phrases matched as literal substrings after stripping and lowercasing; validation telemetry treated the same phrases as prefixes. The reported case-level end point used only the normalized prefix “no acute cardiopulmonary process,” allowing trailing text. It was therefore a narrow target-aligned process measure, supplemented by complete-report recurrence, concentration, lexical diversity, label proxies, and human review. The exact phrases and rules are listed in Text S7 and Table S30 in Multimedia Appendix 1.

Three ON decoding-policy configurations were evaluated (Table 1): ON+B4 used 4-beam search with a repetition penalty of 1.00, ON+G1 used greedy decoding with a penalty of 1.15, and ON+K50 used top-k sampling (k=50) with penalty 1.05. Because the algorithm and penalty varied together, each was analyzed as a complete configuration. No setting-specific filtering or semantic correction was applied after generation.

TokFix applied the same deterministic file-format conversion to every output: it standardized line structure, inserted a header labeled “samples,” removed complete “IMG:” lines, and retained all other report text. It did not rewrite, filter, or semantically correct the reports; full parsing rules are provided in Multimedia Appendix 1.

**Table 1.** Overview of datasets, transfer directions, decoding conditions, and evaluation components.^a^

Category and component	MIMIC^b^→IU	IU→MIMIC	Notes
Domain transfer			
Source training domain	MIMIC-CXR^c^	IU X-ray	Model training domain
Target evaluation domain	IU X-ray	MIMIC-CXR	Cross-domain test domain
Standardized evaluation sample size	575	401	Evaluable outputs entering the fixed audit pipeline
Processing and settings			
Postprocessing	Standardized ValReport conversion	Standardized ValReport conversion	TokFix was used only for standardized formatting and parsability control; it did not perform semantic correction
Main decoding settings in the main text	ON^d^+B4, ON+G1^e^, ON+K50^f^	ON+B4, ON+G1, ON+K50	Main operating profiles compared under the fixed protocol
Audit outputs			
Automated audit outputs	Surface validity; unique-report ratio; exact duplicate rate; top-5 output share; template-prefix rate; Distinct-1/2; CheXpert-based alignment	Surface validity; unique-report ratio; exact duplicate rate; top-5 output share; template-prefix rate; Distinct-1/2; CheXpert-based alignment	Unified layered automated audit
Primary human-audit subset	105 reports	105 reports	35 randomly sampled evaluation cases per direction; 3 reports per case
Targeted failure-mode panel	30 reports	30 reports	10 targeted evaluation cases per direction; descriptive analysis only
Total human-audit workload	135 reports	135 reports	90 evaluation cases and 270 configuration-specific reports; 2 independent reviewers

a Bidirectional cross-domain transfer was evaluated under a fixed audit protocol. TokFix standardized the evaluation-facing file structure without semantic correction. The human audit included 70 randomly sampled primary evaluation cases and 20 targeted failure-mode evaluation cases, with one ON+B4, one ON+G1, and one ON+K50 reported for each case.

b MIMIC: Medical Information Mart for Intensive Care.

c MIMIC-CXR: Medical Information Mart for Intensive Care Chest X-Ray.

d ON: protocol-defined training-side control enabled.

e G1: greedy decoding.

f K50: top-k sampling with k=50.

### Automated Audit Measures

The automated audit combined surface validity, complete-report recurrence and concentration, the predefined prefix, lexical diversity, and Stanford CheXpert/NegBio clinical-label alignment [6,7]. MIMIC→IU outputs were reevaluated without regeneration under the same unified pipeline used for IU→MIMIC.

An output was considered surface-valid when it parsed successfully and remained minimally interpretable as report-like text, without empty content, unresolved special tokens, or structural failure. Rates used the complete evaluation set as the denominator.

Corpus-level concentration was summarized by the unique-report ratio, the proportion of outputs belonging to repeated exact-text groups, and the share represented by the 5 most frequent reports. These measures did not assess case-level correctness.

The predefined prefix rate was the proportion of normalized reports beginning with “no acute cardiopulmonary process,” with subsequent text allowed. Because the phrase also appeared among training keywords, it was a narrow target-aligned end point, not a comprehensive templating measure. The repeated 4-gram rate was 0 in all main runs and was not used for ranking.

Distinct-1 and Distinct-2 were the proportions of unique unigrams and bigrams in each setting-level output collection [29]. They measured surface diversity rather than clinical correctness.

Clinical-content alignment used the Stanford CheXpert labeler (commit 44ddeb363149aa657296237f18b5472a73c1756f) with its Extractor→NegBio Classifier→Aggregator pipeline; NegBio was fixed at commit 073199e2792824740e89844a59c13d3d40ce4d23 [6,7]. All reference and prediction labels reported in the revised analyses were regenerated using the same versioned invocation and mapped back to cases. Across 14 categories, only raw state 1 was positive; raw −1, raw 0, and blank states were mapped to negative. Microprecision, microrecall, and micro-*F*_1_ pooled true positive (TP), false positive (FP), and false negative (FN) across reports and categories; per-label counts and scores were also examined. All were interpreted as labeler-derived proxies.

A seed-based inspection of 20 IU→MIMIC ON+B4 Cardiomegaly-positive outputs and 20 MIMIC→IU ON+B4 outputs prompted a complete text-level audit. Because the initial sample list was not retained, it served only as preliminary quality control.

Primary verification covered all 393 IU→MIMIC ON+B4 outputs labeled “cardiomegaly positive,” including case-reference mapping, final-text provenance, hashes, raw states, binary mapping, and a controlled official-labeler test. The retained predictions and official labels were not altered.

Because this audit identified a cardiomegaly response to recurrent, semantically normal, heart-size wording, a post hoc descriptive sensitivity analysis excluded cardiomegaly from both matrices and recomputed pooled metrics across the remaining 13 categories. No reports or individual labels were removed or corrected, and the analysis was outside the Holm family.

Definitions and interpretive limits for all automated measures are summarized in Table S8 in Multimedia Appendix 1.

### Exploratory MedGemma Stress Test

MedGemma 1.5 4B (revision 91850547d9f0b2fdd21aa7c5f4f3d1a8a52c243b) [30] generated reports without task-specific fine-tuning on the same 575 IU and 401 MIMIC-CXR records; a 553-entry frontal-only IU subset was also analyzed. One fixed prompt and deterministic greedy decoding were used, without prompt search or target-based selection. Because training exposure and generation conditions differed, this was not a matched comparison.

Float16 produced unstable first-token logits and all-padding generations on the RTX 3090 stack, so formal generations were performed using float32. All 976 records yielded nonempty uniquely identified outputs; 512-token-limit outputs were retained and flagged. Reproducibility details and hashes are provided in Multimedia Appendix 1.

MedGemma was evaluated with the same automated definitions. Stand-alone, clustered 95% CIs were calculated using IU unique ID–level clusters and MIMIC-CXR patient clusters and remained outside the primary Holm family. No MedGemma human-review comparison was performed.

### Expanded Human Audit

The blinded human audit included 90 evaluation cases and 270 configuration-specific reports, with one ON+B4, ON+G1, and ON+K50 report per case. The primary random sample comprised 70 cases (35 per direction; 210 reports); a separate targeted panel comprised 20 cases (60 reports) and was excluded from primary proportion summaries. An evaluation case was a linked target record, not necessarily a unique patient.

Primary cases were sampled with seed 2026 without conditioning on validity, template indicators, clinical-label scores, or output quality; the primary and targeted subsets did not overlap. Reports received anonymized identifiers and independently randomized orders. Reviewers were blinded to their configuration, automated results, other configurations, and corpus-wide frequencies.

Two advanced medical trainees from the West China School of Medicine independently reviewed the reports following codebook training and 10 excluded calibration cases. Neither was a licensed radiologist, so the task was treated as structured clinical-content review rather than formal radiological diagnosis.

In phase 1, reviewers saw the radiograph, viewed information, and generated report without the reference; in phase 2, the reference was disclosed for a sensitivity assessment of agreement, omissions, and possible multiview mismatches. The configuration and automated results remained concealed.

Primary end points were major omission; major unsupported finding; 0-to-3 content adequacy and case-specific informativeness; and finding-level TP, FP, and FN counts. Scores ≥2 indicated adequate or informative output. Internal contradiction, unsupported normality, template genericness, reference agreement, and multiview mismatch were secondary or sensitivity measures.

Both reviewers completed all reports before reaching consensus. Protocol-defined disagreements triggered a blinded discussion, and unresolved primary end point disagreements were adjudicated by a third medically trained reviewer who remained blinded. Final summaries used consensus or adjudicated ratings; full rules are provided in Multimedia Appendix 1.

Preconsensus reliability used percent agreement, Cohen κ and Gwet AC1 (agreement coefficient 1) for binary end points, exact and within-1 agreement with quadratically weighted κ for ordinal end points, and intraclass correlation coefficient (ICC) (2,1) with absolute differences for TP, FP, and FN counts.

### Statistical and Descriptive Analysis

Primary automated comparisons used paired cluster bootstrap resampling (5000 replicates; seed 2026) conducted separately by direction, with IU case clusters for MIMIC→IU and MIMIC patient clusters for IU→MIMIC. Within each direction, ON+B4 was compared with OFF+B4, ON+G1, and ON+K50 for surface validity, prefix hit, and CheXpert micro-*F*_1_. Effects were computed as first minus second; 95% CIs and *P* values were Holm-adjusted across 9 tests per direction. Recurrence, concentration, and Distinct-1/2 remained descriptive.

Human-audit summaries used only the 210 reports from the 70 randomly sampled cases and were stratified by direction and configuration; the 60 targeted reports were reserved for qualitative failure analysis.

Binary human end points were reported with exact binomial 95% CIs; ordinal distributions and ≥2 thresholds were reported, and finding-level microprecision, microrecall, and micro-*F*_1_ used pooled consensus counts. No configuration-level human hypothesis tests were performed, so differences were interpreted descriptively.

### Ethical Considerations

This study used 2 previously released deidentified datasets: MIMIC-CXR and IU X-ray [27,28]. MIMIC-CXR is available through credentialed PhysioNet access, required training, and a data use agreement [31]; its release was approved by the Beth Israel Deaconess Medical Center Institutional Review Board with a waiver of consent [27]. IU X-ray was collected with institutional review board approval (Office of Human Subjects Research Protections number 5357) and deidentified before release [28]. The original approval, waiver, and release conditions permitted the secondary research use of the datasets without obtaining additional informed consent from individual patients.

All analyses were performed on deidentified images, deidentified reports, generated text, and derived evaluation outputs. All team members who directly accessed MIMIC-CXR, including reviewers and the adjudicator, held individual authorization, and completed the required training and agreement. Restricted data were processed locally, were not sent to hosted AI services or external APIs, and were not used for reidentification. The authors did not attempt to identify, reidentify, contact, or link any individual patient.

No separate local ethics application was submitted because this secondary analysis involved no recruitment, enrollment, contact, intervention, direct identifiers, new identifiable data, or clinical use of generated reports. Article 32 of the 2023 Chinese Measures for Ethical Review permits a waiver for qualifying research using lawfully obtained public or anonymized data [32]. The authors considered this study to meet those conditions; therefore, no local approval or exemption number was issued and no additional consent was sought.

Because this was a secondary analysis of existing deidentified data, no human participants were recruited, enrolled, contacted, or compensated. The reviewers and adjudicator served as uncompensated research team members, not research participants. No identifiable patient images, facial photographs, names, dates of birth, medical record numbers, accession numbers, or other direct identifiers are included in the paper or Multimedia Appendix 1.

## Results

### Evaluation Cohort Composition and Domain Shift

The MIMIC→IU cohort contained 575 IU entries (553 frontal and 22 lateral fallback inputs). The IU→MIMIC cohort contained 401 frontal MIMIC-CXR entries from 347 studies. No entries were lost due to missing output, failed matching, or configuration-specific evaluability.

The median reference lengths were 34 (IQR 26-47) words for IU and 69 (IQR 49-89) words for MIMIC-CXR. Labeler-derived reference positives also differed: no finding, 33.0% (190/575) vs 9.2% (37/401); lung opacity, 22.3% (128/575) vs 41.9% (168/401); edema, 3.0% (17/575) vs 17.0% (68/401); and support devices, 5.2% (30/575) vs 32.7% (131/401). These are report-label distributions, not patient-level prevalence.

Report length, label composition, view structure, and clustering-contextualized direction-specific findings; within each direction, all configurations used the same fixed entries.

### Overview of Cross-Domain Automated Audit Findings

The primary automated audit jointly assessed validity, recurrence, concentration, the predefined prefix, lexical diversity, and the official CheXpert/NegBio overlap. These end points represented different constructs. MedGemma was assessed separately using the same layers.

Degradation varied by direction and end point, and no ON configuration was uniformly favorable across the automated measures (Tables 2 and 3). The MedGemma results appear separately in Table 4.

**Table 2.** Formal validity, complete-report recurrence, concentration, and protocol-defined prefix results for the 3 ON (protocol-defined training-side control enabled)–controlled decoding policies.^a^

Direction	Configuration	Surface valid, n/N (%)	Unique reports, n/N (%)	Exact duplicate outputs, n/N (%)	Top-5 output share, n/N (%)	Template prefix, n/N (%)
MIMIC^b^→IU	ON^c^+B4^d^	574/575 (99.8)	12/575 (2.1)	569/575 (99.0)	562/575 (97.7)	8/575 (1.4)
MIMIC→IU	ON+G1^e^	574/575 (99.8)	109/575 (19.0)	511/575 (88.9)	325/575 (56.5)	358/575 (62.3)
MIMIC→IU	ON+K50^f^	566/575 (98.4)	246/575 (42.8)	400/575 (69.6)	170/575 (29.6)	222/575 (38.6)
IU→MIMIC	ON+B4	401/401 (100)	37/401 (9.2)	382/401 (95.3)	338/401 (84.3)	0/401 (0)
IU→MIMIC	ON+G1	401/401 (100)	99/401 (24.7)	330/401 (82.3)	276/401 (68.8)	0/401 (0)
IU→MIMIC	ON+K50	401/401 (100)	258/401 (64.3)	208/401 (51.9)	58/401 (14.5)	0/401 (0)

a Exact duplicate rate counts all outputs in repeated complete-report group; top-5 share is the proportion represented by the 5 most frequent reports. The prefix end point is not a global templating measure.

b MIMIC: Medical Information Mart for Intensive Care.

c ON: protocol-defined training-side control enabled.

d B4: beam search with a beam size of 4.

e G1: greedy decoding.

f K50: top-k sampling with k=50.

**Table 3.** Lexical diversity and official clinical-label proxy results for the 3 ON (protocol-defined training-side control enabled)-controlled decoding policies.^a^

Direction	Configuration	Distinct-2	CheXpert micro-*F*_1_
MIMIC→IU^b^	ON+B4^c^	0.0078	0.0608
MIMIC→IU	ON+G1^d^	0.0260	0.1808
MIMIC→IU	ON+K50^e^	0.0495	0.2423
IU→MIMIC	ON+B4	0.0122	0.1687
IU→MIMIC	ON+G1	0.0233	0.1617
IU→MIMIC	ON+K50	0.0691	0.1708

a Distinct-2 measures corpus-level lexical dispersion; CheXpert micro-*F*_1_ is a labeler-derived proxy from the unified Stanford pipeline.

b MIMIC: Medical Information Mart for Intensive Care.

c B4: beam search with a beam size of 4.

d G1: greedy decoding.

e K50: top-k sampling with k=50.

**Table 4.** Exploratory MedGemma 1.5 automated cross-architecture stress-test results.^a^

Metric	IU (n=575)	IU frontal-only (n=553)	MIMIC-CXR^b^ (n=401)
Surface valid	0.9948	0.9946	0.9776
Exact duplicate rate	0.6626	0.6637	0.1446
Top-5 output share	0.4417	0.4467	0.0873
Distinct-1	0.0140	0.0142	0.0173
Distinct-2	0.0505	0.0504	0.0742
Single predefined template prefix rate	0	0	0
Official CheXpert micro-*F*_1_	0.4722	0.4763	0.4340

a MedGemma used a single frozen deterministic configuration outside the primary Holm family. The duplicate rate counts all outputs in repeated complete-report groups. No human review of MedGemma was conducted; full intervals and checks are provided in Multimedia Appendix 1.

b MIMIC-CXR: Medical Information Mart for Intensive Care Chest X-Ray.

In MIMIC→IU, ON+B4 minimized the prefix rate to 8 out of 575 (1.4%) but was most concentrated: 12 (2.1%) reports were unique, 569 (99.0%) belonged to duplicate groups, and the top 5 reports covered 562 (97.7%) outputs. Compared with ON+B4, ON+G1 increased the prefix rate by 0.609 (95% CI 0.567‐0.649; adjusted *P*=.002) and CheXpert micro-*F*_1_ by 0.120 (95% CI 0.087‐0.153; *P*=.002) without changing validity. ON+K50 increased the prefix rate by 0.372 (95% CI 0.330‐0.412; *P*=.002) and micro-*F*_1_ by 0.182 (95% CI 0.144‐0.220; *P*=.002), while reducing validity by 0.014 (95% CI 0.003‐0.026; *P*=.03). ON+B4 had no pathology-positive official labels, and all 24 TPs were no finding; No finding also supplied 126 out of 146 ON+K50 TPs.

In IU→MIMIC, all ON outputs were surface-valid and none met the prefix rule. ON+K50 had the highest unique-report ratio (258/401, 64.3%) and the lowest duplicate rate (208/401, 51.9%) and top-5 share (58/401, 14.5%). Micro-*F*_1_ was 0.1687 for ON+B4, 0.1617 for ON+G1, and 0.1708 for ON+K50; neither G1 nor K50 differed from B4 after Holm correction (adjusted *P*>.99). Of the 102 ON+B4 TPs, 100 were cardiomegaly, while most other categories had no *F*_1_ (Figure 2).

**Figure 2.**
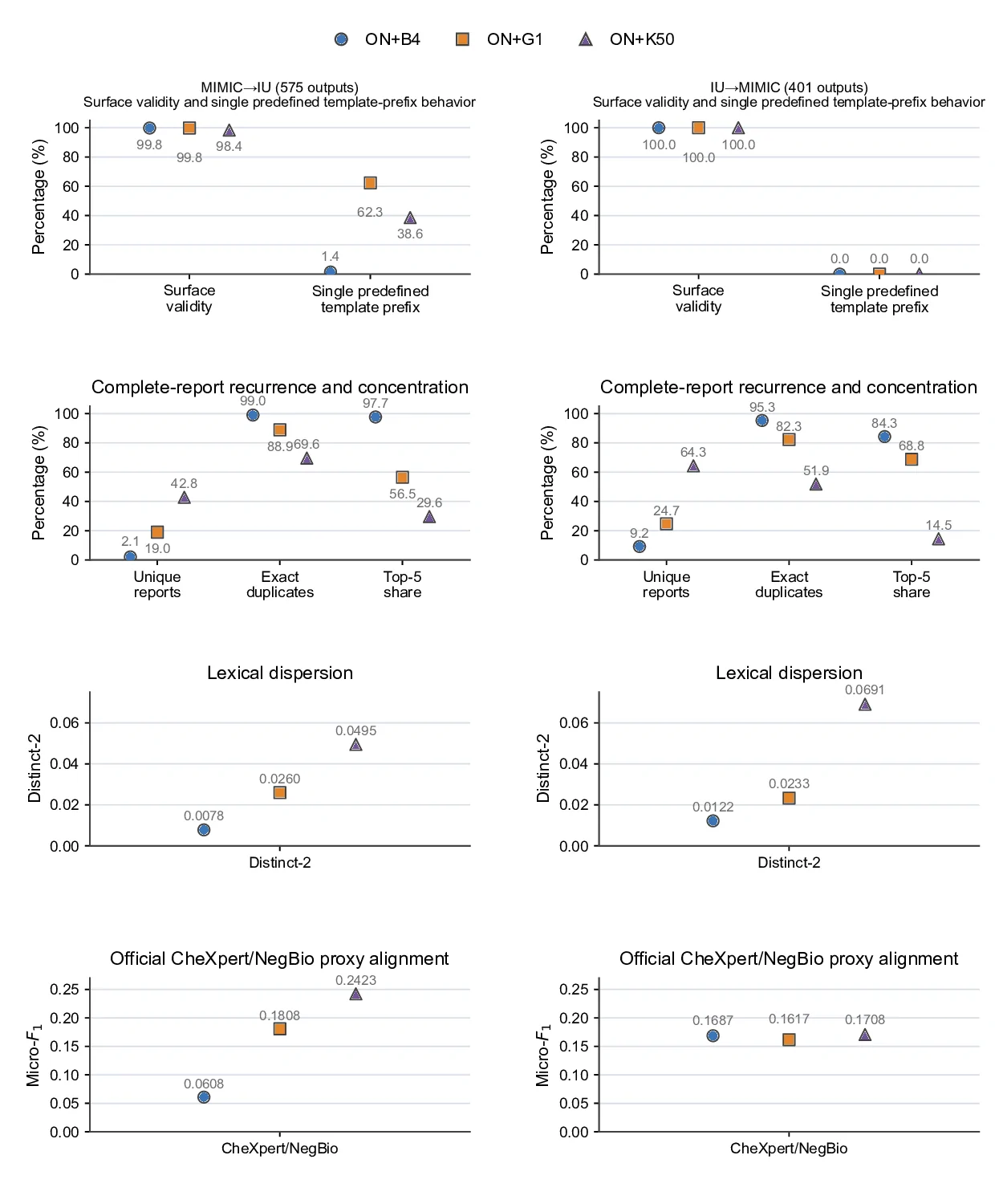
Automated trade-off structure across the main cross-domain decoding settings in the Medical Information Mart for Intensive Care (MIMIC)→IU and IU→MIMIC directions. CheXpert/NegBio micro-*F*_1_ is a labeler-derived proxy. Corpus-level recurrence and diversity measures are descriptive. B4: beam search with a beam size of 4; G1: greedy decoding; K50: top-k sampling with k=50; ON: protocol-defined training-side control enabled.

### Per-Label and Text-Level Audit of the Official CheXpert Proxy

Per-label results were highly concentrated (Table 5). In MIMIC→IU ON+B4, all 13 pathology or device categories had 0 predicted positives; 87 no finding assignments yielded 24 TPs. Although ON+K50 had the highest micro-*F*_1_, no finding supplied 126 out of 146 TPs, and 7 categories plus Support Devices remained at *F*_1_=0.

**Table 5.** Selected per-label official CheXpert/NegBio results illustrating aggregate-score concentration and labeler-response behavior.^a^

Direction/configuration	Observation	Reference positive, n	Predicted positive, n	TP^b^, n	FP^c^, n	FN^d^, n	*F*_1_
MIMIC^e^→IU ON^f^+B4^g^	Cardiomegaly	99	0	0	0	99	0
MIMIC→IU ON+B4	Lung opacity	128	0	0	0	128	0
MIMIC→IU ON+B4	No finding	190	87	24	63	166	0.173
MIMIC→IU ON+K50^h^	Lung opacity	128	3	1	2	127	0.015
MIMIC→IU ON+K50	No finding	190	335	126	209	64	0.480
IU→MIMIC ON+B4	Cardiomegaly	102	393	100	293	2	0.404
IU→MIMIC ON+B4	Lung opacity	168	0	0	0	168	0
IU→MIMIC ON+B4	No finding	37	8	2	6	35	0.089
IU→MIMIC OFF+B4	Cardiomegaly	102	27	9	18	93	0.140
IU→MIMIC OFF+B4	No finding	37	357	31	326	6	0.157

a Values use the uncertain-as-negative policy. Complete 14-label results are given in Tables S24-S27 in Multimedia Appendix 1. IU→MIMIC ON+B4 cardiomegaly positives were predominantly triggered by semantically normal heart-size wording.

b TP: true positive.

c FP: false positive.

d FN: false negative.

e MIMIC: Medical Information Mart for Intensive Care.

f ON: protocol-defined training-side control enabled.

g B4: beam search with a beam size of 4.

h K50: top-k sampling with k=50.

In IU→MIMIC ON+B4, the labeler assigned cardiomegaly positive to 393 of 401 outputs, yielding TP=100, FP=293, FN=2, and TN=6; 100 out of 102 total TPs were obtained from this category. Provenance checks matched all cases and texts across the prediction, label, and metric files. Every positively assigned text used “heart size” with “within normal limits,” and none used “cardiomegaly,” “enlarged,” or “cardiomediastinal”; a controlled official-labeler test also returned cardiomegaly=1.0 for semantically normal wording.

The complete 393-output audit, provenance checks, and controlled test therefore identified a reproducible labeler response that was inconsistent with the apparent sentence meaning, rather than clinically meaningful cardiomegaly generation. Official outputs were retained without relabeling but interpreted as pipeline measurements. MIMIC→IU ON+B4 was similarly described as near-absence of positive label assignments, because some outputs contained fragmented or ambiguous pathology wording despite zero pathology-positive labels.

After excluding cardiomegaly, the IU→MIMIC micro-*F*_1_ was 0.0056 for ON+B4, 0.0666 for OFF+B4, 0.0211 for ON+G1, and 0.0407 for ON+K50. The ON+B4−OFF +B4 difference reversed from +0.0944 to −0.0610 (Table S28 in Multimedia Appendix 1), showing that the 14-category advantage was dominated by the labeler response rather than broad content recovery.

### Training-Control Comparison and Automated Trade-Off Structure

ON/OFF effects varied by direction and end point (Tables 6 and 7). In MIMIC→IU, ON+B4 reduced the prefix rate compared with OFF+B4 by 0.986 (95% CI 0.976‐0.995; adjusted *P*=.002), with no significant validity or micro-*F*_1_ improvement, and remained more concentrated. In IU→MIMIC, validity and prefix rate were identical, while ON+B4 increased 14-category micro-*F*_1_ by 0.094 (95% CI 0.045‐0.155; *P*=.01); per-label and text tracing showed that this contrast was dominated by the normal-heart-size cardiomegaly trigger.

**Table 6.** Unified comparison of ON (protocol-defined training-side control enabled)+B4 (beam search with a beam size of 4) and OFF (protocol-defined training-side control disabled)+B4 under bidirectional cross-domain transfer.^a^

Direction	Condition	Surface valid, n/N (%)	Exact duplicate outputs, n/N (%)	Top-5 output share, n/N (%)	Template prefix, n/N (%)	CheXpert micro-*F*_1_
MIMIC^b^→IU	ON+B4	574/575 (99.8)	569/575 (99.0)	562/575 (97.7)	8/575 (1.4)	0.0608
MIMIC→IU	OFF+B4	575/575 (100)	563/575 (97.9)	517/575 (89.9)	575/575 (100)	0.0687
IU→MIMIC	ON+B4	401/401 (100)	382/401 (95.3)	338/401 (84.3)	0/401 (0)	0.1687
IU→MIMIC	OFF+B4	401/401 (100)	360/401 (89.8)	263/401 (65.6)	0/401 (0)	0.0744

a ON and OFF denote the composite control’s enabled and disabled states under B4 decoding. The contrast does not isolate individual components; recurrence and concentration are descriptive corpus-level measures.

b MIMIC: Medical Information Mart for Intensive Care.

**Table 7.** Primary paired cluster-bootstrap comparisons for the automated audit end points.^a^

Direction and comparison	Surface validity, difference (95% CI)	Adjusted *P*	Template-prefix hit, difference (95% CI)	Adjusted *P*	Official CheXpert micro-*F*_1_, difference (95% CI)	Adjusted *P*
MIMIC^b^→IU: ON^c^+B4^d^−OFF^e^+B4	−0.002 (−0.005 to 0)	>.99	−0.986 (−0.995 to −0.976)	.002	−0.008 (−0.038 to 0.022)	>.99
MIMIC→IU: ON+G1^f^−ON+B4	0.000 (−0.005 to 0.005)	>.99	0.609 (0.567 to 0.649)	.002	0.120 (0.087 to 0.153)	.002
MIMIC→IU: ON+K50^g^−ON+B4	−0.014 (−0.026 to −0.003)	.03	0.372 (0.330 to 0.412)	.002	0.182 (0.144 to 0.220)	.002
IU→MIMIC: ON+B4−OFF+B4	0 (0 to 0)	>.99	0 (0 to 0)	>.99	0.094 (0.045 to 0.155)	.01
IU→MIMIC: ON+G1−ON+B4	0 (0 to 0)	>.99	0 (0 to 0)	>.99	−0.007 (−0.022 to 0.007)	>.99
IU→MIMIC: ON+K50−ON+B4	0 (0 to 0)	>.99	0 (0 to 0)	>.99	0.002 (−0.018 to 0.021)	>.99

a Effects are the first configuration minus the second, with 95% bootstrap CIs and Holm-adjusted *P* values from 5000 replicates (seed 2026). MIMIC→IU used IU case clusters and IU→MIMIC used MIMIC patient clusters. Full estimates and raw *P* values are in Tables S11 to S14 in Multimedia Appendix 1.

b MIMIC: Medical Information Mart for Intensive Care.

c ON: protocol-defined training-side control enabled.

d B4: beam search with a beam size of 4.

e OFF: protocol-defined training-side control disabled.

f G1: greedy decoding.

g K50: top-k sampling with k=50.

Thus, the composite control produced end point–specific changes rather than uniform bidirectional improvement: it suppressed one training-aligned prefix in MIMIC→IU and changed a labeler-derived end point in IU→MIMIC, without consistently reducing complete-report concentration.

### Expanded Human Audit

Both reviewers completed all 270 assessments with complete primary end point linkage. Among 245 items meeting a consensus trigger, 207 were resolved by discussion and 38 by third-reviewer adjudication; all primary end points were resolved. Four items retained missing secondary or sensitivity ratings. The high trigger rate reflected multiple sensitive criteria, not major clinical disagreement on every item.

Consensus summaries showed no consistent configuration ordering (Tables 8 and 9). In IU→MIMIC, ON+G1 had numerically fewer omissions and unsupported findings and micro-*F*_1_ of 0.497, while ON+K50 had the highest unsupported finding rate and the lowest micro-*F*_1_. In MIMIC→IU, ON+B4 and ON+G1 had identical binary error rates; ON+B4 had the highest micro-*F*_1_ (0.529), whereas ON+K50 not only had the most informative reports but also the most omissions and unsupported findings. These patterns were descriptive.

**Table 8.** Primary binary human-audit end points in the randomly sampled evaluation cases (N=35).^a^

Direction and configuration	Major omission, n (%)	Major unsupported finding, n (%)
IU→MIMIC^b^		
ON^c^+B4^d^	15 (43)	12 (34)
ON+G1^e^	12 (34)	10 (29)
ON+K50^f^	13 (37)	15 (43)
MIMIC→IU		
ON+B4	12 (34)	11 (31)
ON+G1	12 (34)	11 (31)
ON+K50	14 (40)	16 (46)

a Values are consensus or adjudicated ratings from the primary random subset; the targeted panel was excluded, and no configuration-level tests were performed.

b MIMIC: Medical Information Mart for Intensive Care.

c ON: protocol-defined training-side control enabled.

d B4: beam search with beam size 4.

e G1: greedy decoding.

f K50: top-k sampling with k=50.

**Table 9.** Human-audit adequacy, informativeness, and finding-level micro-*F*_1_ in the randomly sampled evaluation cases (N=35).^a^

Direction and configuration	Adequacy ≥2, n (%)	Informativeness ≥2, n (%)	Micro-*F*_1_
IU→MIMIC^b^			
ON^c^+B4^d^	16 (46)	15 (43)	0.493
ON+G1^e^	17 (49)	16 (46)	0.497
ON+K50^f^	15 (43)	15 (43)	0.449
MIMIC→IU			
ON+B4	17 (49)	18 (51)	0.529
ON+G1	15 (43)	19 (54)	0.500
ON+K50	16 (46)	20 (57)	0.519

a Scores ≥2 indicated adequate or informative output; micro-*F*_1_ used pooled true positive, false positive, and false negative. No configuration-level tests were performed.

b MIMIC: Medical Information Mart for Intensive Care.

c ON: protocol-defined training-side control enabled.

d B4: beam search with beam size 4.

e G1: greedy decoding.

f K50: top-k sampling with k=50.

Therefore, greater apparent informativeness did not consistently coincide with fewer clinically relevant errors, indicating residual trade-offs among omission, unsupported generation, adequacy, and case specificity. All 35 audited MIMIC→IU ON+B4 reports belonged to repeated complete-report groups (34 in the 5 most frequent groups and 1 in another repeated group). Informativeness ratings assessed whether a report contained image-relevant clinical information for the individual case rather than whether its wording was unique. Nevertheless, 18 out of 35 met the prespecified informativeness threshold: 14 scored 2 and 4 scored 3. Thus, recurrent wording was partially applicable to some cases, but this should not be interpreted as individualized report generation. Finding-level micro-*F*_1_ pooled finding counts and did not penalize the reuse of an identical whole report (Table S40 in Multimedia Appendix 1).

The automated prefix and human genericness end points measured different phenomena. The former detected only the exact opening phrase; the latter assessed the complete report against the image. Prefix-positive reports could contain case-related content, while prefix-negative reports could remain generic or clinically problematic (Tables S38 and S39 in Multimedia Appendix 1).

### Combined Interpretation of Automated and Human Findings

Automated and human measures were complementary. Bootstrap analysis identified direction-specific changes in the target-aligned prefix (adjusted *P*=.002) and the labeler-derived CheXpert end point (adjusted *P*=.01), while per-label tracing exposed the latter’s normal-heart-size cardiomegaly trigger. Human review identified omission, unsupported generation, adequacy, and case specificity not recoverable from those end points alone.

Together, the layers showed multidimensional trade-offs rather than a universal configuration ranking.

Figure 3 summarizes consensus-based human outcomes; full distributions, original ratings, agreement analyses, targeted cases, and comments are provided in Multimedia Appendix 1.

Preconsensus agreement was 78.1% for phase 1 omission (κ=0.543; AC1=0.597) and 80.7% for unsupported findings (κ=0.582; AC1=0.643). Exact agreement was 55.9% for adequacy (weighted κ=0.553) and 51.9% for informativeness (weighted κ=0.487); ICC (2,1) was 0.690 for TP, 0.570 for FP, and 0.530 for FN. Primary end point disagreements were resolved by blinded consensus or adjudication.

**Figure 3.**
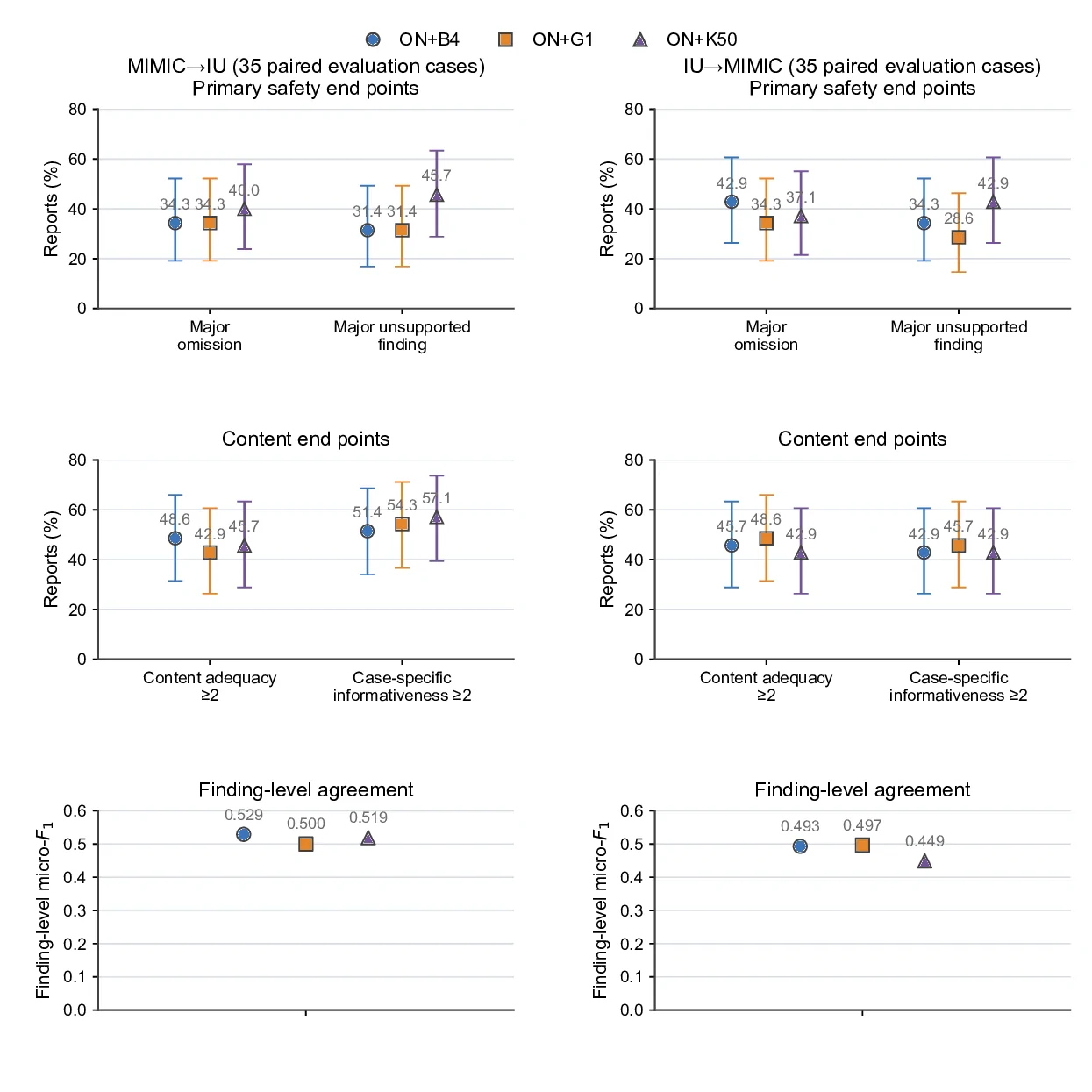
Consensus-based human audit risk profiles across decoding-policy configurations and transfer directions. Points show Consus/adjudicated estimates from the primary random sample. Error bars are Clopper-Pearson exact 95% CIs for report-level proportions. Human audit comparisons are descriptive; no configuration-level hypothesis tests were performed. B4: beam search with beam size 4; G1: greedy decoding; K50: top-k sampling with k=50; MIMIC: Medical Information Mart for Intensive Care; ON: protocol-defined training-side control enabled.

### Exploratory MedGemma Results

MedGemma generated all 976 reports without empty outputs or duplicate identifiers. On IU, surface validity was 0.9948, duplicate rate was 0.6626, top-5 share was 0.4417, Distinct-2 was 0.0505, and micro-*F*_1_ was 0.4722 (95% CI 0.4353‐0.5092); the frontal-only subset was similar. On MIMIC-CXR, the corresponding values were 0.9776, 0.1446, 0.0873, 0.0742, and 0.4340 (95% CI 0.3815‐0.4787).

MedGemma combined higher labeler-derived scores with residual recurrence. In IU, out of 575 outputs, 254 (44.2%) were covered by the top 5 reports and 381 (66.3%) belonged to duplicate groups, despite a 0 % prefix rate, confirming that the narrow prefix did not represent global concentration. Different training conditions and the absence of reader review precluded case-level performance claims.

Four MedGemma reports reached 512 tokens and showed looping or truncation; they were retained and flagged. No MedGemma-generated predictions contained U+FFFD or U+2047 characters. No finding contributed to FPs, whereas MedGemma cardiomegaly FPs generally reflected genuinely positive wording rather than the normal heart-size trigger.

## Discussion

### Principal Results

Cross-domain degradation varied by transfer direction and end point. Neither the composite control nor the 3 decoding policies produced a consistent performance hierarchy. Instead, failures shifted across recurrence, proxy-label alignment, omission, and unsupported generation. The MedGemma stress test further showed that higher label-derived scores could coexist with recurrent output.

### Findings in Relation to the Research Questions

For the first research question, out of 575 outputs, ON reduced the predefined MIMIC→IU prefix from 575 (100%) to 8 (1.4%; adjusted *P*=.002); however, 569 (99.0%) ON+B4 outputs remained duplicates, and the clinical proxy did not improve. In IU→MIMIC, the apparent ON+B4 micro-*F*_1_ advantage was largely a cardiomegaly labeler response to normal heart-size wording and reversed from +0.0944 to −0.0610 after cardiomegaly exclusion. The composite control therefore redistributed, rather than resolved, cross-domain failure.

For the second question, the 3 ON configurations traded prefix frequency, validity, recurrence, diversity, and proxy alignment differently. ON+B4 suppressed the prefix but remained concentrated in MIMIC→IU; ON+K50 increased diversity and reduced concentration but lowered validity and retained prefix recurrence. IU→MIMIC proxy differences were not significant after correction. No policy dominated across end points.

For the third question, out of 35 reports, omissions affected 12 to 15 reports and unsupported findings affected 10 to 16 across the 6 primary cells, with no consistent ordering. Because the human end points were descriptive, these differences did not establish superiority or equivalence.

The ON+G1 results further illustrate that exact-prefix detection and image-grounded whole-report genericness are complementary, not interchangeable, end points.

For the fourth question, MedGemma achieved micro-*F*_1_ values of 0.4722 on IU and 0.4340 on MIMIC-CXR, but 381 out of 575 (66.3%) IU outputs were duplicates. The stress test supports the use of recurrence and label-traceability checks across architectures, not a matched performance or generalizability claim.

### Comparison With Prior Work

Recent studies have advanced structured reporting, interpretable evaluation, reader studies, and disease-aware or foundation-model generators [18-25,30]. In contrast to architecture-focused work by Nimalsiri et al [24] and Edirisinghe et al [25], this study combines symmetric transfer, cluster-aware inference, report concentration, per-label and final-text tracing, and blinded case review.

The cardiomegaly audit illustrates the need to verify reproducible pipeline outputs against the construct they are intended to measure. The comparison of representative prior work is provided in Tables S32 and S33 in Multimedia Appendix 1.

### Implications for Evaluation and Deployment

For deployment beyond source datasets [13-15,17-25,30], suppressing one prefix does not ensure lower report recurrence; aggregate label scores can be driven by a single category or recurrent wording; and diversity can coexist with clinical error. Evaluation should therefore combine distributional, proxy, and blinded image-grounded measures.

Operating configurations should be judged against the intended balance of stability, content retention, case specificity, and error risk. Future work should include more institutions, languages, contemporary models, board-certified radiologists, and prospective workflows, while preserving case-linked provenance and direction-specific inference.

### Limitations

#### Cohort and Data Provenance

The deterministic MIMIC-CXR sample retained 401 of 3374 eligible official-test entries in a fixed row order and may not represent the full eligible cohort. The IU split was verified only at the unique ID level because patient IDs were unavailable; residual patient overlap, if present, could make transfer performance appear better and thus underestimate degradation. Because age and sex were incompletely retained, subgroup performance could not be assessed; accordingly, this study should not be interpreted as providing evidence either for or against algorithmic bias in either transfer direction. In addition, all MIMIC-CXR inputs and most IU inputs were single-projection images, whereas some reference reports summarized multiview examinations; unavailable projections may have contributed to apparent omissions or reference mismatch.

#### Design and Attribution

The primary experiments used one R2Gen-style backbone and 2 deidentified datasets. ON/OFF evaluated a composite protocol without component ablation, and the decoding algorithm and repetition penalty varied together across the ON policies; therefore, individual component or algorithm effects cannot be isolated. The final prefix end point also overlapped with a training keyword and did not establish a global reduction in templating.

#### Proxy Measurement

The cardiomegaly trigger and the concentration of aggregate scores in no Finding or in a single category show that template, diversity, and label-overlap measures cannot replace case-level factual review. The controlled phrase verification was specific to cardiomegaly; whether analogous rule interactions occur in the other 13 CheXpert categories remains unverified. The absence of an observed anomaly should therefore not be interpreted as validation of every label.

#### Human Review

The audit used 35 reports per direction-configuration cell and medically trained students, with a postgraduate trainee adjudicator, rather than board-certified radiologists. It was designed as a structured, descriptive, clinical-content audit, not a definitive reader study, and uncertainty remains around its subgroup estimates.

#### Exploratory Architecture Branch

MedGemma broadened the architectural scope but was assessed only with automated end points, without matched bidirectional training or human review. Its higher labeler-derived micro-*F*_1_ and lower recurrence in some settings cannot establish whether it reduced omission or unsupported generation, rather than improving proxy scores. The persistence of 66.3% exact duplicate membership in IU illustrates that this question remains unresolved.

### Conclusions

Cross-domain report generators failed differently by direction and end point. The composite control reduced one MIMIC→IU prefix, while the apparent IU→MIMIC CheXpert advantage was largely a cardiomegaly labeler response to normal wording. B4, G1, K50, and MedGemma showed distinct validity, recurrence, diversity, label, and human-error profiles. Therefore, robust evaluation requires distributional measures, per-label and final-text verification, and blinded, image-grounded review.

## Supplementary material

10.2196/95888 Multimedia Appendix 1 Expanded human-audit methods, unified automated analyses, cohort construction, implementation details, recent-work comparison, and exploratory MedGemma stress-test results.

## Abbreviations

AC1: Gwet agreement coefficient 1
B4: beam search with a beam size of 4
EOS: end-of-sequence
FN: false negative
FP: false positive
G1: greedy decoding
ICC: intraclass correlation coefficient
K50: top-k sampling with k=50
MIMIC-CXR: Medical Information Mart for Intensive Care Chest X-Ray
OFF: protocol-defined training-side control disabled
ON: protocol-defined training-side control enabled
TP: true positive
