# Supplementary material for: Cross-Domain Chest Radiograph Report Generation Under Dataset Shift: Bidirectional Audit Study
Source: JMIR Med Inform. 2026 Oct 21;14:e95888. doi: 10.2196/95888 (PMC13686446; doi:10.2196/95888)
Supplement: Supplementary file 1 [file medinform-v14-e95888-s001.docx]

**Multimedia Appendix 1. Expanded human-audit methods, unified automated analyses, cohort construction, implementation details, recent-work comparison, and exploratory MedGemma stress-test results**

This appendix provides expanded human-audit methods and results, automated analyses, cohort construction, CheXpert verification, training-control definitions, implementation details, recent-work comparison, and the exploratory MedGemma stress test.

## Expanded human-audit supplementary materials

Table S1. Composition and completion of the expanded human audit^a^

| **Direction** | **Subset** | **Evaluation cases, n** | **Report items, n** | **ON+B4, n** | **ON+G1, n** | **ON+K50, n** | **Analytical role** |
| --- | --- | --- | --- | --- | --- | --- | --- |
|  |  |  |  |  |  |  |  |
| IU→MIMIC | Primary random | 35 | 105 | 35 | 35 | 35 | Primary descriptive human-audit analysis |
| IU→MIMIC | Targeted failure panel | 10 | 30 | 10 | 10 | 10 | Descriptive failure-mode analysis |
| MIMIC→IU | Primary random | 35 | 105 | 35 | 35 | 35 | Primary descriptive human-audit analysis |
| MIMIC→IU | Targeted failure panel | 10 | 30 | 10 | 10 | 10 | Descriptive failure-mode analysis |
| Total | Primary random | 70 | 210 | 70 | 70 | 70 | Main-text descriptive human-audit results |
| Total | Targeted failure panel | 20 | 60 | 20 | 20 | 20 | Supplementary descriptive analysis |
| Total | All reviewed | 90 | 270 | 90 | 90 | 90 | Total review workload |

^a^ Both reviewers completed all 270 reports (540 reviewer-report records) with no missing mandatory ratings and complete blinded-key linkage.

### Appendix Text S1. Human-audit sampling, blinding, and review workflow

The audit comprised 90 evaluation cases and 270 reports, with ON+B4, ON+G1, and ON+K50 outputs linked within each case. The primary random sample comprised 35 cases per direction (70 cases; 210 reports), selected with seed 2026 without reference to automated results or prediction quality. Surface-invalid and template-dominated outputs were retained, and the primary and targeted subsets did not overlap. An evaluation case was a linked target record, not necessarily a unique patient.

The targeted panel added 10 cases per direction (20 cases; 60 reports), selected to enrich for omission, unsupported findings, generic output, cross-configuration disagreement, reference mismatch, or unusual automated indicators. It supported descriptive failure analysis.

Two advanced medical trainees from West China School of Medicine independently reviewed the reports after codebook training and 10 excluded calibration cases. Reviewer A was a senior undergraduate clinical medicine student; Reviewer B was a postgraduate clinical medicine student with chest-radiograph training. Neither was a licensed radiologist. Item order was independently randomized, and configuration, automated metrics, CheXpert labels, and other outputs were concealed.

During phase 1, reviewers saw the radiograph, view information, and generated report but not the reference. During phase 2, the reference was disclosed for sensitivity assessment of agreement, omission, and possible multiview mismatch. Blinding was maintained throughout both phases.

Table S2. Human-audit endpoints and scoring definitions^a^

| **Endpoint** | **Scale** | **Operational definition** | **Analytical role** |
| --- | --- | --- | --- |
|  |  |  |  |
| Major clinically relevant omission | Binary | An important image-supported finding was omitted in a way that could materially affect case interpretation. | Primary safety endpoint |
| Major unsupported finding | Binary | In phase 1, the report introduced an important abnormal finding not supported by the available radiograph and view information. Reference-report context was considered only during the protocol-defined phase 2 sensitivity assessment. | Primary safety endpoint |
| Content adequacy | 0-3 | 0=unacceptable; 1=clearly inadequate or misleading; 2=basically usable but requiring more than minor correction; 3=adequate without a major correction need. | Primary content endpoint; scores ≥2 classified as adequate |
| Case-specific informativeness | 0-3 | 0=almost entirely generic; 1=limited case-specific information; 2=moderately case-specific but incomplete; 3=highly case-specific and sufficiently informative. | Primary content endpoint; scores ≥2 classified as informative |
| Finding-level TP/FP/FN | Nonnegative counts | A finding unit combined observation, polarity, laterality, and location where clinically distinguishable. Minor wording differences were not counted separately. | Primary finding-level endpoint |
| Internal contradiction | Binary | The generated report contained a clinically meaningful internal inconsistency, such as simultaneous affirmation and negation of the same finding. | Secondary safety endpoint |
| Unsupported normality claim | Binary | In phase 1, the report asserted a normal or absent finding not supported by the available radiograph and view information. Reference-report context was considered only during the protocol-defined phase 2 sensitivity assessment. | Secondary safety endpoint |
| Template genericness | 0-2 | 0=no evident template dependence; 1=partly templated; 2=strongly templated or highly generic. | Interpretive endpoint |
| Generated-reference agreement | 0-3 | Reference-aware agreement between the generated and reference reports. | Sensitivity endpoint |
| Possible multiview mismatch | Binary | The reference report may have included information from a view that was unavailable to the model or reviewer during the primary phase. | Sensitivity and quality-control endpoint |

^a^ Phase 1 omission was primary; phase 2 omission was a separate reference-aware sensitivity endpoint.

### Appendix Text S2. Consensus and adjudication procedure

Both reviewers completed all 270 reports independently before any consensus discussion. Original ratings were retained, and the trigger rule was fixed before configuration identities were disclosed.

Consensus was required for disagreement in major omission, major unsupported finding, internal contradiction, or unsupported normality; differences of at least 2 levels in adequacy or informativeness; or differences of at least 2 in TP, FP, or FN counts. Other discrepancies were secondary unless they affected a primary endpoint.

Triggered items were discussed while reviewers remained blinded; unresolved items were adjudicated by a postgraduate clinical medicine trainee who was also blinded. Phase 1 adjudication excluded the reference, whereas phase 2 included it. Final summaries used consensus or adjudicated ratings. Everyone who viewed MIMIC-CXR held individual PhysioNet authorization, completed required training, and signed the data use agreement; restricted data and credentials were not shared. Reviewers and the adjudicator served as uncompensated research-team members.

Table S3. Preconsensus agreement and magnitude of reviewer differences^a^

| **Endpoint** | **Exact agreement, n** | **Exact agreement, %** | **Difference of 1, n** | **Difference ≥2, n** | **Binary disagreement, n** |
| --- | --- | --- | --- | --- | --- |
|  |  |  |  |  |  |
| Phase 1 major omission | 211 | 78.1 | — | — | 59 |
| Phase 2 major omission | 212 | 78.5 | — | — | 58 |
| Major unsupported finding | 218 | 80.7 | — | — | 52 |
| Internal contradiction | 253 | 93.7 | — | — | 17 |
| Unsupported normality claim | 239 | 88.5 | — | — | 31 |
| Possible multiview mismatch | 248 | 91.9 | — | — | 22 |
| Content adequacy | 151 | 55.9 | 81 | 38 | — |
| Case-specific informativeness | 140 | 51.9 | 84 | 46 | — |
| Template genericness | 138 | 51.1 | 91 | 41 | — |
| Generated-reference agreement | 154 | 57.0 | 76 | 40 | — |
| TP count | 204 | 75.6 | 40 | 26 | — |
| FP count | 178 | 65.9 | 54 | 38 | — |
| FN count | 149 | 55.2 | 75 | 46 | — |

^a^ Agreement used the original pre-consensus ratings. The high trigger rate reflected several sensitive rules and was not a reliability statistic.

Table S4. Endpoint-specific preconsensus interreviewer reliability^a^

| **Endpoint** | **Observed agreement, %** | **Primary reliability coefficient** | **Supplementary agreement metric** |
| --- | --- | --- | --- |
|  |  |  |  |
| Phase 1 major omission | 78.1% | Cohen’s κ=0.543 | Gwet’s AC1=0.597 |
| Phase 2 major omission | 78.5% | Cohen’s κ=0.540 | Gwet’s AC1=0.605 |
| Major unsupported finding | 80.7% | Cohen’s κ=0.582 | Gwet’s AC1=0.643 |
| Internal contradiction | 93.7% | Cohen’s κ=0.760 | Gwet’s AC1=0.915 |
| Unsupported normality claim | 88.5% | Cohen’s κ=0.597 | Gwet’s AC1=0.839 |
| Possible multiview mismatch | 91.9% | Cohen’s κ=0.620 | Gwet’s AC1=0.896 |
| Content adequacy | 55.9% exact; 85.9% within 1 level | Quadratic-weighted κ=0.553 | — |
| Case-specific informativeness | 51.9% exact; 83.0% within 1 level | Quadratic-weighted κ=0.487 | — |
| Template genericness | 51.1% exact; 84.8% within 1 level | Quadratic-weighted κ=0.410 | — |
| Generated-reference agreement | 57.0% exact; 85.2% within 1 level | Quadratic-weighted κ=0.580 | — |
| TP count | — | ICC(2,1)=0.690 | Mean absolute difference=0.341 |
| FP count | — | ICC(2,1)=0.570 | Mean absolute difference=0.481 |
| FN count | — | ICC(2,1)=0.530 | Mean absolute difference=0.619 |

^a^ Coefficients used the original ratings: Cohen’s κ and AC1 for binary endpoints, weighted κ for ordinal endpoints, and ICC(2,1) for counts. Reliability reporting remained separate from consensus.

Table S5. Consensus-trigger and adjudication flow^a^

| **Consensus workflow category** | **Report items, n** | **Denominator, n** | **Interpretation** |
| --- | --- | --- | --- |
|  |  |  |  |
| Total reviewed report items | 270 | 270 | All items were independently rated by Reviewer A and Reviewer B |
| Did not trigger consensus | 25 | 270 | No substantive disagreement met a protocol-defined consensus trigger |
| Triggered consensus | 245 | 270 | At least one protocol-defined consensus criterion applied |
| Resolved by reviewer discussion without third-reviewer adjudication | 207 | 245 | Reviewer A and Reviewer B reached consensus through discussion |
| Required third-reviewer adjudication | 38 | 245 | Reviewer discussion did not resolve one or more protocol-defined endpoint disagreements |

^a^ Four items retained unresolved secondary or sensitivity ratings; all primary endpoints were resolved by discussion or adjudication.

### Appendix Text S3. Descriptive analysis of the expanded human audit

The primary sample contained 35 cases and 105 reports per direction; the targeted panel was excluded from primary proportion summaries and configuration ranking.

Binary endpoints used counts, proportions, and exact binomial 95% CIs. Adequacy and informativeness were reported on their 0-to-3 scales and at the ≥2 threshold. Finding-level microprecision, microrecall, and micro-F1 used pooled consensus TP, FP, and FN counts. Pre-consensus reliability used Cohen’s κ and AC1 for binary endpoints, weighted κ for ordinal endpoints, and ICC(2,1) for counts. Final descriptive summaries used consensus or adjudicated ratings.

Human endpoints were summarized descriptively; differences among B4, G1, and K50 were not subjected to configuration-level hypothesis testing. Formal paired inference was confined to the full-cohort automated endpoints.

#### Targeted failure-mode panel

The targeted panel comprised 20 cases and 60 reports and supported descriptive failure analysis. It was excluded from primary summaries and ranking.

## Unified automated audit supplementary materials

Table S6. Formal validity, complete-report recurrence, output concentration, and predefined template-prefix behavior^a^

| **Direction** | **Configuration** | **N** | **Surface valid, n (%)** | **Unique reports, n (%)** | **Exact duplicates, n (%)** | **Top-5 output share, n (%)** | **Template-prefix, n (%)** |
| --- | --- | --- | --- | --- | --- | --- | --- |
|  |  |  |  |  |  |  |  |
| MIMIC→IU | ON+B4 | 575 | 574 (99.8) | 12 (2.1) | 569 (99.0) | 562 (97.7) | 8 (1.4) |
| MIMIC→IU | OFF+B4 | 575 | 575 (100.0) | 26 (4.5) | 563 (97.9) | 517 (89.9) | 575 (100.0) |
| MIMIC→IU | ON+G1 | 575 | 574 (99.8) | 109 (19.0) | 511 (88.9) | 325 (56.5) | 358 (62.3) |
| MIMIC→IU | ON+K50 | 575 | 566 (98.4) | 246 (42.8) | 400 (69.6) | 170 (29.6) | 222 (38.6) |
| IU→MIMIC | ON+B4 | 401 | 401 (100.0) | 37 (9.2) | 382 (95.3) | 338 (84.3) | 0 (0.0) |
| IU→MIMIC | OFF+B4 | 401 | 401 (100.0) | 74 (18.5) | 360 (89.8) | 263 (65.6) | 0 (0.0) |
| IU→MIMIC | ON+G1 | 401 | 401 (100.0) | 99 (24.7) | 330 (82.3) | 276 (68.8) | 0 (0.0) |
| IU→MIMIC | ON+K50 | 401 | 401 (100.0) | 258 (64.3) | 208 (51.9) | 58 (14.5) | 0 (0.0) |

All MIMIC→IU values were unified re-evaluations of retained predictions without regeneration or checkpoint reselection. IU→MIMIC used the clean IU-trained ON and OFF checkpoints, selected without target-domain results. Final runs used one Stanford CheXpert/NegBio pipeline and common definitions. Repeated 4-gram rate was 0; per-label and text audits identified strong No Finding and Cardiomegaly effects.

Table S7. Lexical diversity and Stanford CheXpert/NegBio clinical-content proxy results^a^

| **Direction** | **Configuration** | **Repeated 4-gram rate** | **Distinct-1** | **Distinct-2** | **Microprecision** | **Microrecall** | **Micro-F1** |
| --- | --- | --- | --- | --- | --- | --- | --- |
|  |  |  |  |  |  |  |  |
| MIMIC→IU | ON+B4 | 0.0000 | 0.0057 | 0.0078 | 0.2759 | 0.0341 | 0.0608 |
| MIMIC→IU | OFF+B4 | 0.0000 | 0.0029 | 0.0053 | 0.0665 | 0.0711 | 0.0687 |
| MIMIC→IU | ON+G1 | 0.0000 | 0.0132 | 0.0260 | 0.1782 | 0.1835 | 0.1808 |
| MIMIC→IU | ON+K50 | 0.0000 | 0.0198 | 0.0495 | 0.2908 | 0.2077 | 0.2423 |
| IU→MIMIC | ON+B4 | 0.0000 | 0.0061 | 0.0122 | 0.2544 | 0.1262 | 0.1687 |
| IU→MIMIC | OFF+B4 | 0.0000 | 0.0073 | 0.0182 | 0.1119 | 0.0557 | 0.0744 |
| IU→MIMIC | ON+G1 | 0.0000 | 0.0088 | 0.0233 | 0.2426 | 0.1213 | 0.1617 |
| IU→MIMIC | ON+K50 | 0.0000 | 0.0261 | 0.0691 | 0.2256 | 0.1374 | 0.1708 |

The same re-evaluation procedures, checkpoint provenance, and interpretive considerations apply as in Table S6.

Table S8. Definitions and intended interpretation of the final automated audit measures^a^

| **Metric** | **Computational definition** | **Unit** | **Purpose and limitation** |
| --- | --- | --- | --- |
|  |  |  |  |
| Surface validity | Number and proportion of reports that satisfied the frozen evaluation-facing structural validity checks after TokFix. | Report-level proportion | Identifies structural or evaluation failure; clinical correctness requires separate assessment. |
| Unique-report ratio | Number of distinct normalized complete prediction strings divided by N. | Corpus-level ratio | Lower values indicate stronger complete-report reuse. |
| Exact-duplicate rate | Number of reports in a complete-string group with frequency ≥2, divided by N. | Corpus-level ratio | Includes every report in a duplicated group, and therefore is not equal to 1 minus the unique-report ratio. |
| Top-5 output share | Sum of the frequencies of the 5 most common normalized complete prediction strings divided by N. | Corpus-level ratio | Measures concentration in a small number of dominant outputs. |
| Single predefined template-prefix rate | Number of normalized reports beginning with the exact phrase “no acute cardiopulmonary process” divided by N. | Report-level proportion | Tracks one recurrent, target-aligned prefix rather than overall report templating or clinical quality. |
| Repeated 4-gram rate | Number of reports containing an internally repeated contiguous 4-token sequence divided by N. | Report-level proportion | Supplementary indicator of local recurrence. |
| Distinct-1 | Number of distinct unigrams divided by the total number of generated unigrams in the corpus. | Corpus-level ratio | Measures lexical dispersion rather than factual diversity. |
| Distinct-2 | Number of distinct bigrams divided by the total number of generated bigrams in the corpus. | Corpus-level ratio | Measures lexical dispersion rather than factual diversity. |
| CheXpert microprecision | Total true-positive labels divided by total predicted-positive labels across the evaluated CheXpert observations. | Label-aggregated proxy | Provides coarse clinical-content alignment rather than direct report-level factual verification. |
| CheXpert microrecall | Total true-positive labels divided by total reference-positive labels across the evaluated CheXpert observations. | Label-aggregated proxy | Sensitive to omission of labeled observations. |
| CheXpert micro-F1 | Harmonic mean of CheXpert microprecision and microrecall. | Label-aggregated proxy | Should be considered alongside recurrence, concentration, and human review. |

Recurrence and diversity describe corpus-level distributions, not case-level correctness. Prefix strings and normalization rules were identical across configurations and directions.

Table S9. Descriptive ON+B4 versus OFF+B4 contrasts by transfer direction^a^

| **Direction** | **Endpoint** | **ON+B4** | **OFF+B4** | **ON−OFF difference** | **Audit interpretation** |
| --- | --- | --- | --- | --- | --- |
|  |  |  |  |  |  |
| MIMIC→IU | Single predefined template-prefix rate | 1.4% | 100.0% | −98.6 percentage points | The composite protocol markedly reduced the target-aligned prefix; this does not show an overall reduction in templating. |
| MIMIC→IU | Exact-duplicate rate | 99.0% | 97.9% | +1.1 percentage points | Complete-report recurrence remained extremely high. |
| MIMIC→IU | Top-5 output share | 97.7% | 89.9% | +7.8 percentage points | Output concentration under B4 was not reduced. |
| MIMIC→IU | CheXpert micro-F1 | 0.0608 | 0.0687 | −0.0079 | The automated clinical-content proxy did not improve. |
| IU→MIMIC | Single predefined template-prefix rate | 0.0% | 0.0% | 0.0 percentage points | The target-aligned single-prefix failure was absent in both B4 conditions. |
| IU→MIMIC | Exact-duplicate rate | 95.3% | 89.8% | +5.5 percentage points | Complete-report recurrence was not reduced. |
| IU→MIMIC | Top-5 output share | 84.3% | 65.6% | +18.7 percentage points | Output concentration under B4 was not reduced. |
| IU→MIMIC | CheXpert micro-F1 | 0.1687 | 0.0744 | +0.0944 | The official labeler-derived endpoint increased; however, text tracing showed that the contrast was dominated by a Cardiomegaly response to recurrent, semantically normal wording rather than broad clinical-content recovery. |

These endpoint-specific contrasts did not indicate uniform bidirectional benefit. Paired inference for the 3 primary case-level endpoints is reported in Tables S11-S14.

Table S10. Provenance and analytical role of the final automated result rows^a^

| **Direction** | **Configuration** | **Prediction/checkpoint provenance** | **Evaluation package or run** | **Analytical role** |
| --- | --- | --- | --- | --- |
|  |  |  |  |  |
| MIMIC→IU | ON+B4 | Retained configuration 4 prediction file; unified evaluation rerun only | M2I_UNIFIED_OFFICIAL_EVAL | Primary ON beam evidence |
| MIMIC→IU | OFF+B4 | Retained configuration 4 prediction file; unified evaluation rerun only | M2I_UNIFIED_OFFICIAL_EVAL | Training-control reference |
| MIMIC→IU | ON+G1 | Retained configuration 4 prediction file; unified evaluation rerun only | M2I_UNIFIED_OFFICIAL_EVAL | ON deterministic policy profile |
| MIMIC→IU | ON+K50 | Retained configuration 4 prediction file; unified evaluation rerun only | M2I_UNIFIED_OFFICIAL_EVAL | ON stochastic policy profile |
| IU→MIMIC | ON+B4 | Clean IU-trained checkpoint 4f9d2fa; fixed decoding seed 2026 | revision_iu2m_clean_retrain/02_fixed_decoding | Primary ON beam evidence |
| IU→MIMIC | OFF+B4 | Clean IU-OFF checkpoint selected by the IU validation BLEU-4 score | IU2M_OFF_B4_CLEAN_RERUN/analysis | Training-control reference |
| IU→MIMIC | ON+G1 | Clean IU-trained checkpoint 4f9d2fa; fixed decoding seed 2026 | revision_iu2m_clean_retrain/02_fixed_decoding | ON deterministic policy profile |
| IU→MIMIC | ON+K50 | Clean IU-trained checkpoint 4f9d2fa; fixed decoding seed 2026 | revision_iu2m_clean_retrain/02_fixed_decoding | ON stochastic policy profile |

This table separates the final unified results from retired CheXbert, chex_v2, and other non-unified values.

Table S11. MIMIC-to-IU endpoint estimates for paired cluster-bootstrap comparisons^a^

| **Comparison** | **Endpoint** | **Estimate A** | **Estimate B** |
| --- | --- | --- | --- |
|  |  |  |  |
| ON+B4 − OFF+B4 | Surface validity | 0.9983 | 1.0000 |
| ON+B4 − OFF+B4 | Template-prefix hit | 0.0139 | 1.0000 |
| ON+B4 − OFF+B4 | CheXpert micro-F1 | 0.0608 | 0.0687 |
| ON+G1 − ON+B4 | Surface validity | 0.9983 | 0.9983 |
| ON+G1 − ON+B4 | Template-prefix hit | 0.6226 | 0.0139 |
| ON+G1 − ON+B4 | CheXpert micro-F1 | 0.1808 | 0.0608 |
| ON+K50 − ON+B4 | Surface validity | 0.9843 | 0.9983 |
| ON+K50 − ON+B4 | Template-prefix hit | 0.3861 | 0.0139 |
| ON+K50 − ON+B4 | CheXpert micro-F1 | 0.2423 | 0.0608 |

Estimates A and B correspond to the named configurations; MIMIC→IU resampling used 575 IU case clusters.

Table S12. MIMIC-to-IU paired differences and multiplicity-adjusted inference^a^

| **Comparison** | **Endpoint** | **Difference (95% CI)** | **Raw P** | **Holm-adjusted P** |
| --- | --- | --- | --- | --- |
|  |  |  |  |  |
| ON+B4 − OFF+B4 | Surface validity | −0.0017 (−0.0052 to 0) | .744 | >.99 |
| ON+B4 − OFF+B4 | Template-prefix hit | −0.9861 (−0.9948 to −0.9757) | <.001 | .002 |
| ON+B4 − OFF+B4 | CheXpert micro-F1 | −0.0080 (−0.0377 to 0.0221) | .593 | >.99 |
| ON+G1 − ON+B4 | Surface validity | 0.0000 (−0.0052 to 0.0052) | >.99 | >.99 |
| ON+G1 − ON+B4 | Template-prefix hit | 0.6087 (0.5670 to 0.6487) | <.001 | .002 |
| ON+G1 − ON+B4 | CheXpert micro-F1 | 0.1200 (0.0870 to 0.1534) | <.001 | .002 |
| ON+K50 − ON+B4 | Surface validity | −0.0139 (−0.0261 to −0.0035) | .007 | .027 |
| ON+K50 − ON+B4 | Template-prefix hit | 0.3722 (0.3304 to 0.4122) | <.001 | .002 |
| ON+K50 − ON+B4 | CheXpert micro-F1 | 0.1816 (0.1436 to 0.2200) | <.001 | .002 |

Differences are A minus B. Paired bootstrap used 5000 replicates and seed 2026; CheXpert micro-F1 was recalculated from pooled counts, and Holm correction covered 9 tests per direction. Corpus-level measures had no case-level P values. Source files are listed in the statistical manifest.

Table S13. IU-to-MIMIC endpoint estimates for paired cluster-bootstrap comparisons^a^

| **Comparison** | **Endpoint** | **Estimate A** | **Estimate B** |
| --- | --- | --- | --- |
|  |  |  |  |
| ON+B4 − OFF+B4 | Surface validity | 1.0000 | 1.0000 |
| ON+B4 − OFF+B4 | Template-prefix hit | 0.0000 | 0.0000 |
| ON+B4 − OFF+B4 | CheXpert micro-F1 | 0.1687 | 0.0744 |
| ON+G1 − ON+B4 | Surface validity | 1.0000 | 1.0000 |
| ON+G1 − ON+B4 | Template-prefix hit | 0.0000 | 0.0000 |
| ON+G1 − ON+B4 | CheXpert micro-F1 | 0.1617 | 0.1687 |
| ON+K50 − ON+B4 | Surface validity | 1.0000 | 1.0000 |
| ON+K50 − ON+B4 | Template-prefix hit | 0.0000 | 0.0000 |
| ON+K50 − ON+B4 | CheXpert micro-F1 | 0.1708 | 0.1687 |

Estimates A and B correspond to the named configurations; IU→MIMIC resampling used MIMIC patient clusters for 401 outputs.

Table S14. IU-to-MIMIC paired differences and multiplicity-adjusted inference^a^

| **Comparison** | **Endpoint** | **Difference (95% CI)** | **Raw P** | **Holm-adjusted P** |
| --- | --- | --- | --- | --- |
|  |  |  |  |  |
| ON+B4 − OFF+B4 | Surface validity | 0.0000 (0 to 0) | >.99 | >.99 |
| ON+B4 − OFF+B4 | Template-prefix hit | 0.0000 (0 to 0) | >.99 | >.99 |
| ON+B4 − OFF+B4 | CheXpert micro-F1 | 0.0944 (0.0445 to 0.1551) | .001 | .011 |
| ON+G1 − ON+B4 | Surface validity | 0.0000 (0 to 0) | >.99 | >.99 |
| ON+G1 − ON+B4 | Template-prefix hit | 0.0000 (0 to 0) | >.99 | >.99 |
| ON+G1 − ON+B4 | CheXpert micro-F1 | −0.0070 (−0.0220 to 0.0066) | .311 | >.99 |
| ON+K50 − ON+B4 | Surface validity | 0.0000 (0 to 0) | >.99 | >.99 |
| ON+K50 − ON+B4 | Template-prefix hit | 0.0000 (0 to 0) | >.99 | >.99 |
| ON+K50 − ON+B4 | CheXpert micro-F1 | 0.0020 (−0.0184 to 0.0206) | .815 | >.99 |

Differences are A minus B. Paired bootstrap used 5000 replicates and seed 2026; CheXpert micro-F1 was recalculated from pooled counts, and Holm correction covered 9 tests per direction. Corpus-level measures had no case-level P values. Source files are listed in the statistical manifest.

## Implementation details

Table S15. Full training hyperparameters^a^

| **Component** | **Setting** |
| --- | --- |
|  |  |
| Optimizer | Adam |
| Initial learning rate | 5×10^-5 |
| Scheduler | StepLR |
| Epochs | 10 |
| Batch size | 8 |
| num_workers | 2 |
| Visual encoder | OpenAI CLIP ViT-B/32 |
| Visual branch | Frozen |
| Visual features | Cached, read-only |
| Precision mode | Mixed fp32/tf32 |
| Tokenization | SentencePiece |
| Maximum generation length | 100 |
| Image setting | MIMIC-CXR: frontal-only; IU X-Ray: frontal preferred, with first-available lateral fallback when no frontal projection was available (22/575 final IU test entries) |

^a^ Shared base hyperparameters are shown here. Tables S29-S31 define the active composite contrast and distinguish the training keywords from the final prefix endpoint.

Table S16. Full decoding hyperparameters^a^

| **Setting** | **Decode method** | **Beam / Top-k / Top-p** | **Repetition penalty** | **No-repeat n-gram** | **Length penalty** | **Seed** |
| --- | --- | --- | --- | --- | --- | --- |
|  |  |  |  |  |  |  |
| ON+B4 | Beam search | num_beams=4 | 1.00 | 3 | 1.0 | 2026 |
| ON+G1 | Greedy | Not applicable | 1.15 | 3 | Not applicable | 2026 |
| ON+K50 | Top-k sampling | top_k=50, top_p=1.0 | 1.05 | 3 | Not applicable | 2026 |

^a^ B4, G1, and K50 were complete configurations because the decoding algorithm and repetition penalty varied together. Runs used seed 2026 without setting-specific filtering or semantic postprocessing.

### Appendix Text S4. TokFix protocol and implementation notes

TokFix standardized report containers before evaluation. It used universal line splitting, wrote a header labeled “samples”, removed complete IMG: lines, normalized line endings, and otherwise preserved each line.

Downstream parsing began after the “samples” header, skipped lines beginning with #, extracted GT: and PR: lines by prefix removal, and paired them in order. TokFix did not filter samples, repair punctuation or sections, rewrite empty reports, or alter clinical content; final audits found no identifier or prediction-text mismatch.

The same sequence of operations was applied to every configuration and direction before the validity, recurrence, concentration, prefix, diversity, and CheXpert/NegBio analyses.

Table S17. Consensus-based primary human-audit safety, adequacy, and informativeness results^a^

| **Direction** | **Configuration** | **N** | **Major omission, n (%)** | **Unsupported finding, n (%)** | **Internal contradiction, n (%)** | **Adequacy ≥2, n (%)** | **Informativeness ≥2, n (%)** |
| --- | --- | --- | --- | --- | --- | --- | --- |
|  |  |  |  |  |  |  |  |
| IU→MIMIC | ON+B4 | 35 | 15 (43) | 12 (34) | 5 (14) | 16 (46) | 15 (43) |
| IU→MIMIC | ON+G1 | 35 | 12 (34) | 10 (29) | 7 (20) | 17 (49) | 16 (46) |
| IU→MIMIC | ON+K50 | 35 | 13 (37) | 15 (43) | 6 (17) | 15 (43) | 15 (43) |
| MIMIC→IU | ON+B4 | 35 | 12 (34) | 11 (31) | 5 (14) | 17 (49) | 18 (51) |
| MIMIC→IU | ON+G1 | 35 | 12 (34) | 11 (31) | 3 (9) | 15 (43) | 19 (54) |
| MIMIC→IU | ON+K50 | 35 | 14 (40) | 16 (46) | 4 (11) | 16 (46) | 20 (57) |

^a^ Consensus or adjudicated primary-random-subset ratings; N=35 per configuration and direction. The targeted panel was excluded.

Table S18. Consensus-based primary human-audit finding-level results^a^

| **Direction** | **Configuration** | **TP, n** | **FP, n** | **FN, n** | **Microprecision** | **Microrecall** | **Micro-F1** |
| --- | --- | --- | --- | --- | --- | --- | --- |
|  |  |  |  |  |  |  |  |
| IU→MIMIC | ON+B4 | 33 | 37 | 31 | 0.471 | 0.516 | 0.493 |
| IU→MIMIC | ON+G1 | 38 | 39 | 38 | 0.494 | 0.500 | 0.497 |
| IU→MIMIC | ON+K50 | 35 | 42 | 44 | 0.455 | 0.443 | 0.449 |
| MIMIC→IU | ON+B4 | 36 | 34 | 30 | 0.514 | 0.545 | 0.529 |
| MIMIC→IU | ON+G1 | 40 | 38 | 42 | 0.513 | 0.488 | 0.500 |
| MIMIC→IU | ON+K50 | 41 | 39 | 37 | 0.512 | 0.526 | 0.519 |

^a^ Microprecision, microrecall, and micro-F1 were calculated from pooled TP, FP, and FN counts.

Table S19. Exact binomial 95% confidence intervals for primary human-audit proportions^a^

| **Direction** | **Configuration** | **Metric** | **n/N** | **Rate, %** | **Exact 95% CI** |
| --- | --- | --- | --- | --- | --- |
|  |  |  |  |  |  |
| IU→MIMIC | ON+B4 | Major omission | 15/35 | 43 | 26.3-60.6 |
| IU→MIMIC | ON+B4 | Unsupported finding | 12/35 | 34 | 19.1-52.2 |
| IU→MIMIC | ON+B4 | Adequacy ≥2 | 16/35 | 46 | 28.8-63.4 |
| IU→MIMIC | ON+B4 | Informativeness ≥2 | 15/35 | 43 | 26.3-60.6 |
| IU→MIMIC | ON+G1 | Major omission | 12/35 | 34 | 19.1-52.2 |
| IU→MIMIC | ON+G1 | Unsupported finding | 10/35 | 29 | 14.6-46.3 |
| IU→MIMIC | ON+G1 | Adequacy ≥2 | 17/35 | 49 | 31.4-66.0 |
| IU→MIMIC | ON+G1 | Informativeness ≥2 | 16/35 | 46 | 28.8-63.4 |
| IU→MIMIC | ON+K50 | Major omission | 13/35 | 37 | 21.5-55.1 |
| IU→MIMIC | ON+K50 | Unsupported finding | 15/35 | 43 | 26.3-60.6 |
| IU→MIMIC | ON+K50 | Adequacy ≥2 | 15/35 | 43 | 26.3-60.6 |
| IU→MIMIC | ON+K50 | Informativeness ≥2 | 15/35 | 43 | 26.3-60.6 |
| MIMIC→IU | ON+B4 | Major omission | 12/35 | 34 | 19.1-52.2 |
| MIMIC→IU | ON+B4 | Unsupported finding | 11/35 | 31 | 16.9-49.3 |
| MIMIC→IU | ON+B4 | Adequacy ≥2 | 17/35 | 49 | 31.4-66.0 |
| MIMIC→IU | ON+B4 | Informativeness ≥2 | 18/35 | 51 | 34.0-68.6 |
| MIMIC→IU | ON+G1 | Major omission | 12/35 | 34 | 19.1-52.2 |
| MIMIC→IU | ON+G1 | Unsupported finding | 11/35 | 31 | 16.9-49.3 |
| MIMIC→IU | ON+G1 | Adequacy ≥2 | 15/35 | 43 | 26.3-60.6 |
| MIMIC→IU | ON+G1 | Informativeness ≥2 | 19/35 | 54 | 36.6-71.2 |
| MIMIC→IU | ON+K50 | Major omission | 14/35 | 40 | 23.9-57.9 |
| MIMIC→IU | ON+K50 | Unsupported finding | 16/35 | 46 | 28.8-63.4 |
| MIMIC→IU | ON+K50 | Adequacy ≥2 | 16/35 | 46 | 28.8-63.4 |
| MIMIC→IU | ON+K50 | Informativeness ≥2 | 20/35 | 57 | 39.4-73.7 |

^a^ Clopper-Pearson intervals describe uncertainty within each 35-case cell and do not provide between-configuration inference.

## Cohort construction and domain-shift supplementary materials

### Appendix Text S5. Prepared cohort construction, split integrity, and analytical scope

The MIMIC-CXR preparation flow was fully reconstructed from the official test split through final evaluation. Figure S1 and Table S22 report the row counts, predefined exclusions, deterministic cap, split integrity, and final completeness checks.

prepare_iuxray.py produced 3826 eligible IU entries, split at the UID level with seed 42. The 575-entry test set had no UID overlap across partitions; reliable patient identifiers were unavailable, so patient-level disjointness was not independently verified.

For MIMIC-CXR, 5159 official-test rows were scanned in fixed metadata order. The predefined PA/AP/AP SUPINE filter retained 3403 rows. No report files were missing; 29 reports were excluded because cleaned Impression+Findings text contained fewer than 32 characters; and no image files were missing, leaving 3374 eligible image-level entries. The predetermined cap retained the first 401 entries (11.9%), representing 347 studies. CheXpert labels were metadata rather than filters, and no random seed or output-based selection was used.

All MIMIC-CXR inputs were frontal. The IU cohort comprised 553 frontal and 22 lateral fallback entries, selected under the fixed preparation rule when no frontal projection was available.

After test partitions were fixed, all 575 IU and 401 MIMIC-CXR entries had images, references, complete outputs for all 4 configurations, and successful case-reference alignment; no post hoc attrition occurred.

Table S20. Characteristics of the locally prepared source-domain and final target-domain cohorts^a^

| **Characteristic** | **MIMIC-CXR source/training** | **IU X-Ray source/training** | **MIMIC-CXR final test** | **IU X-Ray final test** |
| --- | --- | --- | --- | --- |
|  |  |  |  |  |
| Patients, n | 887 | NA—not available in the prepared IU metadata | Not reported in this analysis | NA—not reliably available |
| Studies or prepared study entries, n | 2920 | 2678 | 347 | 575 |
| Image entries, n | 3198 | 2678 | 401 | 575 |
| Report-bearing entries, n | 3198 | 2678 | 401 | 575 |
| Unique normalized report texts, n | 2850 | 2189 | 347 | 522 |
| Mean images per study | 1.10 | 1.00 | 1.16 | 1.00 |
| Frontal images, n (%) | 3198 (100.0%) | 2569 (95.9%) | 401 (100.0%) | 553 (96.2%) |
| Lateral images, n (%) | 0 (0.0%) | 109 (4.1%) | 0 (0.0%) | 22 (3.8%) |
| Mean report length, words | 57.0 | 37.9 | 71.1 | 39.3 |
| Median report length, words (interquartile range) | 52 (37–70) | 34 (25–46) | 69 (49–89) | 34 (26–47) |
| Mean number of sentences | 6.31 | 6.17 | 7.04 | 6.22 |
| Empty prepared reports, n (%) | 0 (0.0%) | 0 (0.0%) | 0 (0.0%) | 0 (0.0%) |
| Demographic fields | Not exported in the prepared annotations | Not reliably available | Not exported in the prepared annotations | Not reliably available |

These statistics describe the locally prepared project annotations, not the full public releases. Unique normalized reports are exact deduplicated texts, and demographic comparisons were unavailable because required fields were not reliably retained.

Table S21. Official CheXpert/NegBio reference-report observation-label distribution in the final target cohorts^a^

| **Observation** | **MIMIC final test positive, n/N (%)** | **IU final test positive, n/N (%)** | **Uncertain labels, n (%)** |
| --- | --- | --- | --- |
|  |  |  |  |
| Atelectasis | 81/401 (20.2%) | 52/575 (9.0%) | MIMIC 24 (6.0%); IU 12 (2.1%) |
| Cardiomegaly | 102/401 (25.4%) | 99/575 (17.2%) | MIMIC 14 (3.5%); IU 7 (1.2%) |
| Consolidation | 30/401 (7.5%) | 32/575 (5.6%) | MIMIC 11 (2.7%); IU 4 (0.7%) |
| Edema | 68/401 (17.0%) | 17/575 (3.0%) | MIMIC 39 (9.7%); IU 18 (3.1%) |
| Enlarged Cardiomediastinum | 27/401 (6.7%) | 24/575 (4.2%) | MIMIC 27 (6.7%); IU 37 (6.4%) |
| Fracture | 4/401 (1.0%) | 18/575 (3.1%) | MIMIC 1 (0.2%); IU 1 (0.2%) |
| Lung Lesion | 12/401 (3.0%) | 33/575 (5.7%) | MIMIC 0 (0.0%); IU 1 (0.2%) |
| Lung Opacity | 168/401 (41.9%) | 128/575 (22.3%) | MIMIC 11 (2.7%); IU 10 (1.7%) |
| No Finding | 37/401 (9.2%) | 190/575 (33.0%) | MIMIC 0 (0.0%); IU 0 (0.0%) |
| Pleural Effusion | 74/401 (18.5%) | 36/575 (6.3%) | MIMIC 6 (1.5%); IU 20 (3.5%) |
| Pleural Other | 13/401 (3.2%) | 5/575 (0.9%) | MIMIC 4 (1.0%); IU 0 (0.0%) |
| Pneumonia | 52/401 (13.0%) | 22/575 (3.8%) | MIMIC 51 (12.7%); IU 8 (1.4%) |
| Pneumothorax | 9/401 (2.2%) | 17/575 (3.0%) | MIMIC 3 (0.7%); IU 19 (3.3%) |
| Support Devices | 131/401 (32.7%) | 30/575 (5.2%) | MIMIC 1 (0.2%); IU 0 (0.0%) |

Labels were extracted from the final references using the Stanford pipeline and the uncertain-as-negative policy. They represent documented observations rather than patient-level disease prevalence.

Figure S1. Reconstructed cohort flow. The MIMIC-CXR path shows all counts from 5159 official-test rows to 3374 eligible entries and the deterministic first-401 cap; the IU path shows the UID-level split and view composition. Cohorts were fixed before decoding and human audit, with no configuration-specific attrition.

Table S22. Cohort traceability, split integrity, and final-evaluation completeness^a^

| **Item** | **MIMIC→IU (IU target)** | **IU→MIMIC (MIMIC target)** |
| --- | --- | --- |
|  |  |  |
| Prepared split construction | 3826 eligible entries; IU UID-level split; seed 42 | 5159 official-test rows → 3403 frontal-view rows → 3374 eligible entries; fixed row order; no random seed |
| Overlap audit | No IU UID overlap across train, validation, and test | Official MIMIC split assignments retained; final clustering based on subject_id; CheXpert labels not used for filtering |
| Prepared test size | 575 entries | 3374 eligible entries before cap; first 401 retained (11.9%); 347 studies |
| View composition | 553 frontal; 22 lateral fallback | 401 frontal |
| Available reference reports | 575/575 | 401/401 |
| Available image inputs | 575/575 | 401/401 |
| Complete outputs for 4 configurations | 575/575 per configuration | 401/401 per configuration |
| Successful case-reference matching | 575/575 | 401/401 |
| Final automatic evaluation | 575 entries | 401 entries |
| Selection timing and independence | Split fixed before model training and evaluation | Predetermined cap applied during annotation construction before training/decoding/evaluation; no seed, output-, audit-, or label-based selection |

The deterministic MIMIC-CXR row-order subset was independent of outputs and labels, although retaining 401/3374 eligible entries may reduce representativeness. IU non-overlap was verified at the UID level because patient identifiers were unavailable; any residual patient overlap would likely make cross-domain performance appear better than it actually is and therefore underestimate transfer degradation.

### Appendix Text S6. Official CheXpert per-label and text-level sanity audit

Complete 14-label results used Stanford chexpert-labeler commit 44ddeb363149aa657296237f18b5472a73c1756f with the Extractor→NegBio Classifier→Aggregator pipeline and NegBio commit 073199e2792824740e89844a59c13d3d40ce4d23. All reference and prediction labels reported in the revised analyses were regenerated under this unified pipeline using the same direction-specific versioned calls and mapped back to cases. Under the uncertain-as-negative policy, raw 1 was positive, whereas raw −1, raw 0, and blank states were negative. The per-label tables report reference and prediction positives, TP, FP, FN, precision, recall, and F1 without post hoc relabeling.

An initial seed-based inspection of 20 IU→MIMIC ON+B4 Cardiomegaly-positive outputs and 20 MIMIC→IU ON+B4 outputs prompted a complete audit. As the original sample list had not been retained, the primary evidence comprised the full 393-report phrase audit, file linkage, raw-state and mapping checks, and a controlled official-labeler test. No report, model, or checkpoint was altered.

For IU→MIMIC ON+B4, predicted Cardiomegaly states were 393 positive and 8 negative; under the primary mapping, TP=100, FP=293, FN=2, TN=6, precision=0.2545, recall=0.9804, and F1=0.4040. All 393 positive-assigned texts contained “heart size” and “within normal limits”; none contained explicit enlargement terms, and a controlled normal-heart-size sentence also returned Cardiomegaly=1.0. The result was attributed to a reproducible labeler response rather than clinically meaningful cardiomegaly generation.

For MIMIC→IU ON+B4, 87/575 outputs were labeled No Finding positive and all other categories had zero predicted positives. Some low-diversity texts contained fragmented or ambiguous pathology wording, so this indicates near-absence of pathology-positive label assignments rather than absence of all pathology terminology. The controlled phrase verification was specific to Cardiomegaly; whether analogous rule interactions occur in the other 13 categories remains unverified, and those labels remain proxies rather than verified clinical judgments.

Field-flow checks found no identifier errors in either direction and no prediction-text mismatches across 1604 IU→MIMIC configuration rows. For ON+B4, all 401 cases, texts, references, images, label-map entries, and metric records matched, establishing computational traceability while revealing limitations in the labeling pipeline.

The CheXpert labeler worktree was clean. In the NegBio worktree, the only modified files were PLY-generated parser.out and parsetab.py caches rebuilt under PLY 3.11 from tables originally serialized by PLY 3.10. Grammar productions, source rules, patterns, actions, and goto behavior were unchanged; static table comparison showed equivalent parsing after state renumbering. The run package retained per-sample labels, text maps, automated metrics, per-label and confusion files, and recorded SHA-256 hashes.

Table S23. Text-level and raw-state verification of outlying official CheXpert cells

| **Audit item** | **Result** | **Interpretation** |
| --- | --- | --- |
|  |  |  |
| IU→MIMIC ON+B4 raw Cardiomegaly states | 393 raw 1.0; 8 raw 0.0 | No binary-mapping error |
| Positive-state wording | 393/393 contained “heart size” and “within normal limits” | Recurrent semantically normal wording |
| Explicit abnormal terms | 0/393 contained “cardiomegaly,” “enlarged,” or “cardiomediastinal” | Not literal cardiomegaly generation |
| Single-case official-labeler test | Normal heart-size sentence returned raw Cardiomegaly=1.0 | Direct labeler-facing trigger |
| IU→MIMIC field flow | 0 identifier or prediction-text mismatches | Final generated text used |
| MIMIC→IU ON+B4 labels | 87 No Finding; 0 positive in other 13 categories | Near-absence under uncertain-as-negative |
| Model/checkpoint changes | None | Analytical verification only |
| Independent confusion reconstruction | TP=100; FP=293; FN=2; TN=6; precision/recall/F1=0.2545/0.9804/0.4040 | Matches the raw per-sample and per-label summaries |
| End-to-end file linkage | 401/401 IDs, predictions, references, and image paths matched across final generation and evaluation artifacts | No configuration, field, or text mismatch |
| Seeded 20-case review asset | Seed documented in the review report; no standalone case-list file retained | Full 393-report phrase audit and single-case test provide the primary reproducibility support |

Table S24. Complete official CheXpert/NegBio per-label reference, prediction, and confusion counts for MIMIC-to-IU^a^

| **Configuration** | **Observation** | **Reference positive, n** | **Predicted positive, n** | **TP, n** | **FP, n** | **FN, n** |
| --- | --- | --- | --- | --- | --- | --- |
|  |  |  |  |  |  |  |
| ON+B4 | Atelectasis | 52 | 0 | 0 | 0 | 52 |
| ON+B4 | Cardiomegaly | 99 | 0 | 0 | 0 | 99 |
| ON+B4 | Consolidation | 32 | 0 | 0 | 0 | 32 |
| ON+B4 | Edema | 17 | 0 | 0 | 0 | 17 |
| ON+B4 | Enlarged Cardiomediastinum | 24 | 0 | 0 | 0 | 24 |
| ON+B4 | Fracture | 18 | 0 | 0 | 0 | 18 |
| ON+B4 | Lung Lesion | 33 | 0 | 0 | 0 | 33 |
| ON+B4 | Lung Opacity | 128 | 0 | 0 | 0 | 128 |
| ON+B4 | No Finding | 190 | 87 | 24 | 63 | 166 |
| ON+B4 | Pleural Effusion | 36 | 0 | 0 | 0 | 36 |
| ON+B4 | Pleural Other | 5 | 0 | 0 | 0 | 5 |
| ON+B4 | Pneumonia | 22 | 0 | 0 | 0 | 22 |
| ON+B4 | Pneumothorax | 17 | 0 | 0 | 0 | 17 |
| ON+B4 | Support Devices | 30 | 0 | 0 | 0 | 30 |
| OFF+B4 | Atelectasis | 52 | 0 | 0 | 0 | 52 |
| OFF+B4 | Cardiomegaly | 99 | 26 | 6 | 20 | 93 |
| OFF+B4 | Consolidation | 32 | 295 | 22 | 273 | 10 |
| OFF+B4 | Edema | 17 | 0 | 0 | 0 | 17 |
| OFF+B4 | Enlarged Cardiomediastinum | 24 | 383 | 18 | 365 | 6 |
| OFF+B4 | Fracture | 18 | 0 | 0 | 0 | 18 |
| OFF+B4 | Lung Lesion | 33 | 0 | 0 | 0 | 33 |
| OFF+B4 | Lung Opacity | 128 | 0 | 0 | 0 | 128 |
| OFF+B4 | No Finding | 190 | 5 | 1 | 4 | 189 |
| OFF+B4 | Pleural Effusion | 36 | 0 | 0 | 0 | 36 |
| OFF+B4 | Pleural Other | 5 | 0 | 0 | 0 | 5 |
| OFF+B4 | Pneumonia | 22 | 0 | 0 | 0 | 22 |
| OFF+B4 | Pneumothorax | 17 | 43 | 3 | 40 | 14 |
| OFF+B4 | Support Devices | 30 | 0 | 0 | 0 | 30 |
| ON+G1 | Atelectasis | 52 | 111 | 10 | 101 | 42 |
| ON+G1 | Cardiomegaly | 99 | 139 | 21 | 118 | 78 |
| ON+G1 | Consolidation | 32 | 0 | 0 | 0 | 32 |
| ON+G1 | Edema | 17 | 14 | 0 | 14 | 17 |
| ON+G1 | Enlarged Cardiomediastinum | 24 | 2 | 0 | 2 | 24 |
| ON+G1 | Fracture | 18 | 0 | 0 | 0 | 18 |
| ON+G1 | Lung Lesion | 33 | 0 | 0 | 0 | 33 |
| ON+G1 | Lung Opacity | 128 | 1 | 1 | 0 | 127 |
| ON+G1 | No Finding | 190 | 283 | 86 | 197 | 104 |
| ON+G1 | Pleural Effusion | 36 | 27 | 4 | 23 | 32 |
| ON+G1 | Pleural Other | 5 | 0 | 0 | 0 | 5 |
| ON+G1 | Pneumonia | 22 | 90 | 5 | 85 | 17 |
| ON+G1 | Pneumothorax | 17 | 18 | 2 | 16 | 15 |
| ON+G1 | Support Devices | 30 | 39 | 0 | 39 | 30 |
| ON+K50 | Atelectasis | 52 | 48 | 8 | 40 | 44 |
| ON+K50 | Cardiomegaly | 99 | 29 | 6 | 23 | 93 |
| ON+K50 | Consolidation | 32 | 1 | 0 | 1 | 32 |
| ON+K50 | Edema | 17 | 19 | 1 | 18 | 16 |
| ON+K50 | Enlarged Cardiomediastinum | 24 | 5 | 0 | 5 | 24 |
| ON+K50 | Fracture | 18 | 0 | 0 | 0 | 18 |
| ON+K50 | Lung Lesion | 33 | 0 | 0 | 0 | 33 |
| ON+K50 | Lung Opacity | 128 | 3 | 1 | 2 | 127 |
| ON+K50 | No Finding | 190 | 335 | 126 | 209 | 64 |
| ON+K50 | Pleural Effusion | 36 | 46 | 4 | 42 | 32 |
| ON+K50 | Pleural Other | 5 | 0 | 0 | 0 | 5 |
| ON+K50 | Pneumonia | 22 | 4 | 0 | 4 | 22 |
| ON+K50 | Pneumothorax | 17 | 4 | 0 | 4 | 17 |
| ON+K50 | Support Devices | 30 | 8 | 0 | 8 | 30 |

^a^ Positive labels followed the uncertain-as-negative policy. Precision, recall, and F1 used standard pooled-count formulae; zero denominators were reported as 0. The ontology excludes Infiltrate.

Table S25. Complete official CheXpert/NegBio per-label precision, recall, and F1 for MIMIC-to-IU^a^

| **Configuration** | **Observation** | **Precision** | **Recall** | **F1** |
| --- | --- | --- | --- | --- |
|  |  |  |  |  |
| ON+B4 | Atelectasis | 0.000 | 0.000 | 0.000 |
| ON+B4 | Cardiomegaly | 0.000 | 0.000 | 0.000 |
| ON+B4 | Consolidation | 0.000 | 0.000 | 0.000 |
| ON+B4 | Edema | 0.000 | 0.000 | 0.000 |
| ON+B4 | Enlarged Cardiomediastinum | 0.000 | 0.000 | 0.000 |
| ON+B4 | Fracture | 0.000 | 0.000 | 0.000 |
| ON+B4 | Lung Lesion | 0.000 | 0.000 | 0.000 |
| ON+B4 | Lung Opacity | 0.000 | 0.000 | 0.000 |
| ON+B4 | No Finding | 0.276 | 0.126 | 0.173 |
| ON+B4 | Pleural Effusion | 0.000 | 0.000 | 0.000 |
| ON+B4 | Pleural Other | 0.000 | 0.000 | 0.000 |
| ON+B4 | Pneumonia | 0.000 | 0.000 | 0.000 |
| ON+B4 | Pneumothorax | 0.000 | 0.000 | 0.000 |
| ON+B4 | Support Devices | 0.000 | 0.000 | 0.000 |
| OFF+B4 | Atelectasis | 0.000 | 0.000 | 0.000 |
| OFF+B4 | Cardiomegaly | 0.231 | 0.061 | 0.096 |
| OFF+B4 | Consolidation | 0.075 | 0.688 | 0.135 |
| OFF+B4 | Edema | 0.000 | 0.000 | 0.000 |
| OFF+B4 | Enlarged Cardiomediastinum | 0.047 | 0.750 | 0.088 |
| OFF+B4 | Fracture | 0.000 | 0.000 | 0.000 |
| OFF+B4 | Lung Lesion | 0.000 | 0.000 | 0.000 |
| OFF+B4 | Lung Opacity | 0.000 | 0.000 | 0.000 |
| OFF+B4 | No Finding | 0.200 | 0.005 | 0.010 |
| OFF+B4 | Pleural Effusion | 0.000 | 0.000 | 0.000 |
| OFF+B4 | Pleural Other | 0.000 | 0.000 | 0.000 |
| OFF+B4 | Pneumonia | 0.000 | 0.000 | 0.000 |
| OFF+B4 | Pneumothorax | 0.070 | 0.176 | 0.100 |
| OFF+B4 | Support Devices | 0.000 | 0.000 | 0.000 |
| ON+G1 | Atelectasis | 0.090 | 0.192 | 0.123 |
| ON+G1 | Cardiomegaly | 0.151 | 0.212 | 0.176 |
| ON+G1 | Consolidation | 0.000 | 0.000 | 0.000 |
| ON+G1 | Edema | 0.000 | 0.000 | 0.000 |
| ON+G1 | Enlarged Cardiomediastinum | 0.000 | 0.000 | 0.000 |
| ON+G1 | Fracture | 0.000 | 0.000 | 0.000 |
| ON+G1 | Lung Lesion | 0.000 | 0.000 | 0.000 |
| ON+G1 | Lung Opacity | 1.000 | 0.008 | 0.016 |
| ON+G1 | No Finding | 0.304 | 0.453 | 0.364 |
| ON+G1 | Pleural Effusion | 0.148 | 0.111 | 0.127 |
| ON+G1 | Pleural Other | 0.000 | 0.000 | 0.000 |
| ON+G1 | Pneumonia | 0.056 | 0.227 | 0.089 |
| ON+G1 | Pneumothorax | 0.111 | 0.118 | 0.114 |
| ON+G1 | Support Devices | 0.000 | 0.000 | 0.000 |
| ON+K50 | Atelectasis | 0.167 | 0.154 | 0.160 |
| ON+K50 | Cardiomegaly | 0.207 | 0.061 | 0.094 |
| ON+K50 | Consolidation | 0.000 | 0.000 | 0.000 |
| ON+K50 | Edema | 0.053 | 0.059 | 0.056 |
| ON+K50 | Enlarged Cardiomediastinum | 0.000 | 0.000 | 0.000 |
| ON+K50 | Fracture | 0.000 | 0.000 | 0.000 |
| ON+K50 | Lung Lesion | 0.000 | 0.000 | 0.000 |
| ON+K50 | Lung Opacity | 0.333 | 0.008 | 0.015 |
| ON+K50 | No Finding | 0.376 | 0.663 | 0.480 |
| ON+K50 | Pleural Effusion | 0.087 | 0.111 | 0.098 |
| ON+K50 | Pleural Other | 0.000 | 0.000 | 0.000 |
| ON+K50 | Pneumonia | 0.000 | 0.000 | 0.000 |
| ON+K50 | Pneumothorax | 0.000 | 0.000 | 0.000 |
| ON+K50 | Support Devices | 0.000 | 0.000 | 0.000 |

^a^ Definitions and mapping follow Table S24.

Table S26. Complete official CheXpert/NegBio per-label reference, prediction, and confusion counts for IU-to-MIMIC^a^

| **Configuration** | **Observation** | **Reference positive, n** | **Predicted positive, n** | **TP, n** | **FP, n** | **FN, n** |
| --- | --- | --- | --- | --- | --- | --- |
|  |  |  |  |  |  |  |
| ON+B4 | Atelectasis | 81 | 0 | 0 | 0 | 81 |
| ON+B4 | Cardiomegaly | 102 | 393 | 100 | 293 | 2 |
| ON+B4 | Consolidation | 30 | 0 | 0 | 0 | 30 |
| ON+B4 | Edema | 68 | 0 | 0 | 0 | 68 |
| ON+B4 | Enlarged Cardiomediastinum | 27 | 0 | 0 | 0 | 27 |
| ON+B4 | Fracture | 4 | 0 | 0 | 0 | 4 |
| ON+B4 | Lung Lesion | 12 | 0 | 0 | 0 | 12 |
| ON+B4 | Lung Opacity | 168 | 0 | 0 | 0 | 168 |
| ON+B4 | No Finding | 37 | 8 | 2 | 6 | 35 |
| ON+B4 | Pleural Effusion | 74 | 0 | 0 | 0 | 74 |
| ON+B4 | Pleural Other | 13 | 0 | 0 | 0 | 13 |
| ON+B4 | Pneumonia | 52 | 0 | 0 | 0 | 52 |
| ON+B4 | Pneumothorax | 9 | 0 | 0 | 0 | 9 |
| ON+B4 | Support Devices | 131 | 0 | 0 | 0 | 131 |
| OFF+B4 | Atelectasis | 81 | 0 | 0 | 0 | 81 |
| OFF+B4 | Cardiomegaly | 102 | 27 | 9 | 18 | 93 |
| OFF+B4 | Consolidation | 30 | 1 | 0 | 1 | 30 |
| OFF+B4 | Edema | 68 | 1 | 1 | 0 | 67 |
| OFF+B4 | Enlarged Cardiomediastinum | 27 | 1 | 0 | 1 | 27 |
| OFF+B4 | Fracture | 4 | 0 | 0 | 0 | 4 |
| OFF+B4 | Lung Lesion | 12 | 1 | 0 | 1 | 12 |
| OFF+B4 | Lung Opacity | 168 | 13 | 4 | 9 | 164 |
| OFF+B4 | No Finding | 37 | 357 | 31 | 326 | 6 |
| OFF+B4 | Pleural Effusion | 74 | 1 | 0 | 1 | 74 |
| OFF+B4 | Pleural Other | 13 | 0 | 0 | 0 | 13 |
| OFF+B4 | Pneumonia | 52 | 0 | 0 | 0 | 52 |
| OFF+B4 | Pneumothorax | 9 | 0 | 0 | 0 | 9 |
| OFF+B4 | Support Devices | 131 | 0 | 0 | 0 | 131 |
| ON+G1 | Atelectasis | 81 | 0 | 0 | 0 | 81 |
| ON+G1 | Cardiomegaly | 102 | 353 | 90 | 263 | 12 |
| ON+G1 | Consolidation | 30 | 2 | 0 | 2 | 30 |
| ON+G1 | Edema | 68 | 0 | 0 | 0 | 68 |
| ON+G1 | Enlarged Cardiomediastinum | 27 | 4 | 0 | 4 | 27 |
| ON+G1 | Fracture | 4 | 0 | 0 | 0 | 4 |
| ON+G1 | Lung Lesion | 12 | 0 | 0 | 0 | 12 |
| ON+G1 | Lung Opacity | 168 | 2 | 1 | 1 | 167 |
| ON+G1 | No Finding | 37 | 42 | 7 | 35 | 30 |
| ON+G1 | Pleural Effusion | 74 | 1 | 0 | 1 | 74 |
| ON+G1 | Pleural Other | 13 | 0 | 0 | 0 | 13 |
| ON+G1 | Pneumonia | 52 | 0 | 0 | 0 | 52 |
| ON+G1 | Pneumothorax | 9 | 0 | 0 | 0 | 9 |
| ON+G1 | Support Devices | 131 | 0 | 0 | 0 | 131 |
| ON+K50 | Atelectasis | 81 | 0 | 0 | 0 | 81 |
| ON+K50 | Cardiomegaly | 102 | 362 | 94 | 268 | 8 |
| ON+K50 | Consolidation | 30 | 10 | 2 | 8 | 28 |
| ON+K50 | Edema | 68 | 0 | 0 | 0 | 68 |
| ON+K50 | Enlarged Cardiomediastinum | 27 | 58 | 3 | 55 | 24 |
| ON+K50 | Fracture | 4 | 0 | 0 | 0 | 4 |
| ON+K50 | Lung Lesion | 12 | 1 | 0 | 1 | 12 |
| ON+K50 | Lung Opacity | 168 | 13 | 8 | 5 | 160 |
| ON+K50 | No Finding | 37 | 27 | 3 | 24 | 34 |
| ON+K50 | Pleural Effusion | 74 | 12 | 1 | 11 | 73 |
| ON+K50 | Pleural Other | 13 | 0 | 0 | 0 | 13 |
| ON+K50 | Pneumonia | 52 | 3 | 0 | 3 | 52 |
| ON+K50 | Pneumothorax | 9 | 6 | 0 | 6 | 9 |
| ON+K50 | Support Devices | 131 | 0 | 0 | 0 | 131 |

^a^ Definitions and mapping follow Table S24.

Table S27. Complete official CheXpert/NegBio per-label precision, recall, and F1 for IU-to-MIMIC^a^

| **Configuration** | **Observation** | **Precision** | **Recall** | **F1** |
| --- | --- | --- | --- | --- |
|  |  |  |  |  |
| ON+B4 | Atelectasis | 0.000 | 0.000 | 0.000 |
| ON+B4 | Cardiomegaly | 0.254 | 0.980 | 0.404 |
| ON+B4 | Consolidation | 0.000 | 0.000 | 0.000 |
| ON+B4 | Edema | 0.000 | 0.000 | 0.000 |
| ON+B4 | Enlarged Cardiomediastinum | 0.000 | 0.000 | 0.000 |
| ON+B4 | Fracture | 0.000 | 0.000 | 0.000 |
| ON+B4 | Lung Lesion | 0.000 | 0.000 | 0.000 |
| ON+B4 | Lung Opacity | 0.000 | 0.000 | 0.000 |
| ON+B4 | No Finding | 0.250 | 0.054 | 0.089 |
| ON+B4 | Pleural Effusion | 0.000 | 0.000 | 0.000 |
| ON+B4 | Pleural Other | 0.000 | 0.000 | 0.000 |
| ON+B4 | Pneumonia | 0.000 | 0.000 | 0.000 |
| ON+B4 | Pneumothorax | 0.000 | 0.000 | 0.000 |
| ON+B4 | Support Devices | 0.000 | 0.000 | 0.000 |
| OFF+B4 | Atelectasis | 0.000 | 0.000 | 0.000 |
| OFF+B4 | Cardiomegaly | 0.333 | 0.088 | 0.140 |
| OFF+B4 | Consolidation | 0.000 | 0.000 | 0.000 |
| OFF+B4 | Edema | 1.000 | 0.015 | 0.029 |
| OFF+B4 | Enlarged Cardiomediastinum | 0.000 | 0.000 | 0.000 |
| OFF+B4 | Fracture | 0.000 | 0.000 | 0.000 |
| OFF+B4 | Lung Lesion | 0.000 | 0.000 | 0.000 |
| OFF+B4 | Lung Opacity | 0.308 | 0.024 | 0.044 |
| OFF+B4 | No Finding | 0.087 | 0.838 | 0.157 |
| OFF+B4 | Pleural Effusion | 0.000 | 0.000 | 0.000 |
| OFF+B4 | Pleural Other | 0.000 | 0.000 | 0.000 |
| OFF+B4 | Pneumonia | 0.000 | 0.000 | 0.000 |
| OFF+B4 | Pneumothorax | 0.000 | 0.000 | 0.000 |
| OFF+B4 | Support Devices | 0.000 | 0.000 | 0.000 |
| ON+G1 | Atelectasis | 0.000 | 0.000 | 0.000 |
| ON+G1 | Cardiomegaly | 0.255 | 0.882 | 0.396 |
| ON+G1 | Consolidation | 0.000 | 0.000 | 0.000 |
| ON+G1 | Edema | 0.000 | 0.000 | 0.000 |
| ON+G1 | Enlarged Cardiomediastinum | 0.000 | 0.000 | 0.000 |
| ON+G1 | Fracture | 0.000 | 0.000 | 0.000 |
| ON+G1 | Lung Lesion | 0.000 | 0.000 | 0.000 |
| ON+G1 | Lung Opacity | 0.500 | 0.006 | 0.012 |
| ON+G1 | No Finding | 0.167 | 0.189 | 0.177 |
| ON+G1 | Pleural Effusion | 0.000 | 0.000 | 0.000 |
| ON+G1 | Pleural Other | 0.000 | 0.000 | 0.000 |
| ON+G1 | Pneumonia | 0.000 | 0.000 | 0.000 |
| ON+G1 | Pneumothorax | 0.000 | 0.000 | 0.000 |
| ON+G1 | Support Devices | 0.000 | 0.000 | 0.000 |
| ON+K50 | Atelectasis | 0.000 | 0.000 | 0.000 |
| ON+K50 | Cardiomegaly | 0.260 | 0.922 | 0.405 |
| ON+K50 | Consolidation | 0.200 | 0.067 | 0.100 |
| ON+K50 | Edema | 0.000 | 0.000 | 0.000 |
| ON+K50 | Enlarged Cardiomediastinum | 0.052 | 0.111 | 0.071 |
| ON+K50 | Fracture | 0.000 | 0.000 | 0.000 |
| ON+K50 | Lung Lesion | 0.000 | 0.000 | 0.000 |
| ON+K50 | Lung Opacity | 0.615 | 0.048 | 0.088 |
| ON+K50 | No Finding | 0.111 | 0.081 | 0.094 |
| ON+K50 | Pleural Effusion | 0.083 | 0.014 | 0.023 |
| ON+K50 | Pleural Other | 0.000 | 0.000 | 0.000 |
| ON+K50 | Pneumonia | 0.000 | 0.000 | 0.000 |
| ON+K50 | Pneumothorax | 0.000 | 0.000 | 0.000 |
| ON+K50 | Support Devices | 0.000 | 0.000 | 0.000 |

^a^ Definitions and mapping follow Table S24.

Table S28. Post hoc 13-category CheXpert sensitivity analysis excluding Cardiomegaly for IU-to-MIMIC^a^

| **Configuration** | **Categories, n** | **TP, n** | **FP, n** | **FN, n** | **Microprecision** | **Microrecall** | **Micro-F1** |
| --- | --- | --- | --- | --- | --- | --- | --- |
|  |  |  |  |  |  |  |  |
| ON+B4 | 13 | 2 | 6 | 704 | 0.2500 | 0.0028 | 0.0056 |
| OFF+B4 | 13 | 36 | 339 | 670 | 0.0960 | 0.0510 | 0.0666 |
| ON+G1 | 13 | 8 | 43 | 698 | 0.1569 | 0.0113 | 0.0211 |
| ON+K50 | 13 | 17 | 113 | 689 | 0.1308 | 0.0241 | 0.0407 |

Cardiomegaly was removed from both matrices for all 401 IU→MIMIC records, without removing reports or manually correcting labels. With the uncertain-as-negative mapping unchanged, the ON+B4−OFF+B4 micro-F1 difference was −0.0610 rather than +0.0944. This descriptive sensitivity analysis remained outside the Holm family.

## Training-control reproducibility supplement

### Appendix Text S7. Composite training-side control and final template-prefix endpoint

Audit of the code and configuration confirmed that ON enabled non-template-balanced sampling, lag-1 and lag-2 recurrence penalties, early-EOS suppression, EOS-token cross-entropy reweighting, and a template-token probability penalty; OFF used the default cross-entropy path. The adaptive controller, late-EOS term, and tail recurrence multiplier were inactive.

The implemented objective was L_ON = CE + 0.9×CE_EOS + 0.02×UL_1 + 0.01×UL_2 + 0.02×EOS_early + 0.08×TemplatePenalty. UL_1 penalized the previous target token, UL_2 was a lag-2 recurrence proxy, and the template penalty covered 16 positions beginning at token 32. OFF used default CE with minimum target length 24.

Training marking used the following 6 lowercase literal phrases: “no acute cardiopulmonary process”; “no acute intrathoracic process”; “no acute cardiopulmonary abnormality”; “no evidence of acute cardiopulmonary”; “no active cardiopulmonary disease”; and “lungs are clear” (all shown without terminal punctuation). Reports and keywords were stripped and lowercased; training marking used substring matching anywhere, whereas validation telemetry used prefix matching. The reported endpoint used only the normalized prefix “no acute cardiopulmonary process,” with trailing text allowed. Table S30 distinguishes these stages. The final endpoint therefore measured one target-aligned failure, not all templating, recurrence, or clinical quality.

In the IU training split (N=2678), 1177 reports were template marked and 1501 were not. The ON sampler targeted a non-template ratio of 0.96 (floor 0.94) independently across 8 ranks. Cross-rank duplication was possible, and the realized unique epoch composition was not reconstructed. Validation and test flags were descriptive.

A parallel audit found the same default phrase list, matching functions, active ON components, and OFF defaults for MIMIC-CXR source runs, with shared base settings held constant within each ON/OFF comparison. The experiments therefore support protocol-level rather than component-specific attribution.

Table S29. Active ON/OFF training-control components and implementation granularity^a^

| **Component** | **ON setting** | **OFF setting** | **Granularity** | **Implementation role** | **Interpretive note** |
| --- | --- | --- | --- | --- | --- |
|  |  |  |  |  |  |
| Non-template sampler | Enabled; target 0.96, floor 0.94 | Disabled | Training sample/report | Changes epoch sampling composition | Nominal ratio only; the audited IU run used no within-rank replacement, although cross-rank duplication was possible. |
| Lag-1 recurrence penalty | λ=0.02 | 0 | Token position | Penalises p_t(y_{t-1}) | Local recurrence proxy |
| Lag-2 recurrence proxy | λ=0.01 | 0 | Token position | Penalises p_t(y_{t-2}) | Not a strict joint bigram objective |
| Early-EOS suppression | λ=0.02; L_min=32 | 0; default L_min=24 | Token position | Penalises early EOS probability | Late-EOS encouragement was inactive |
| EOS CE reweighting | Scale 1.9; added coefficient 0.9 | Scale 1.0 | EOS target positions | Upweights eligible EOS CE | Part of composite protocol |
| Template-token probability penalty | Weight 0.08; 16 positions from token 32 | 0 | Token-position window | Penalises probability mass on template-keyword token IDs | No semantic correction is performed |
| Adaptive controller | Off | Off | Validation telemetry | No adaptive ratio or penalty update | Inactive |
| Tail recurrence multiplier | 1.0 | 1.0 | Token position | No tail-specific increase | Inactive |

^a^ Settings were verified against the clean ON/OFF implementation; training, decoding, labeling, and bootstrap analyses were not rerun.

Table S30. Distinction among training-side marking, validation telemetry, and the final reported prefix endpoint^a^

| **Stage** | **Phrase set** | **Normalization** | **Match location** | **Aggregation** | **Analytical role** |
| --- | --- | --- | --- | --- | --- |
|  |  |  |  |  |  |
| Training sample marking | 6 frozen phrases listed in Appendix Text S7 | strip + lowercase; no internal whitespace or punctuation normalization | Substring anywhere; no regex | One Boolean template flag per sample | Sampler eligibility |
| Validation telemetry | Same 6 phrases | strip + lowercase | Prefix | Setting-level rate | Training monitoring |
| Final main endpoint | 1 phrase: no acute cardiopulmonary process | strip + lowercase; final evaluation normalization applied | Case-insensitive normalized prefix; trailing text allowed | One Boolean flag per case | Target-aligned process endpoint |

^a^ Phrase sets, normalization, match locations, and aggregation levels were verified against the audited code; analyses were not rerun.

Table S31. Interpretive scope of the ON/OFF contrast^a^

| **Evidence** | **Supported interpretation** | **Interpretive boundary** |
| --- | --- | --- |
|  |  |  |
| ON versus OFF under B4 | Effect of the composite training protocol within each transfer direction | Causal contribution of any individual component |
| Single-prefix endpoint | Change in one frozen, target-aligned recurrent prefix | An overall reduction in report templating |
| Official CheXpert endpoint | Reproducible difference in a labeler-derived proxy | Direct evidence of broad clinical correctness |
| Corpus recurrence/diversity | Setting-level output concentration and lexical dispersion | Independent case-level clinical performance |
| Expanded human audit | Case-level omission, unsupported findings, adequacy, and informativeness within the audited subset | Universal superiority of any decoding policy |

^a^ Claims were checked against clean training, configuration, data-loader, loss, and metric code; analyses were not rerun.

## Recent prior-work positioning supplement

### Appendix Text S8. Scope of the targeted recent-work update

A targeted update through 5 July 2026 included representative published or accepted 2023-2026 studies and one official model card spanning generation, structured outputs, evaluation, error detection, and reader studies. The present study differs by combining symmetric transfer, direction-stratified inference, corpus-level degeneration, final-text provenance, and independent review. MedGemma remained exploratory.

Table S32. Recent prior work: study focus, data setting, directionality, and evaluation granularity^a^

| **Study / status** | **Primary focus** | **Data setting** | **Symmetric bidirectional transfer** | **Evaluation granularity** |
| --- | --- | --- | --- | --- |
|  |  |  |  |  |
| Delbrouck et al, 2025[1]; ACL | Structured report task; SRR-BERT / F1-SRR-BERT | MIMIC-CXR; CheXpert Plus | No | 55-label structured taxonomy |
| RadEval, 2025[2]; EMNLP demo | Unified open-source evaluation framework | Multiple public radiology text datasets | No | Lexical, embedding, clinical-concept, and LLM metrics |
| ICARE, 2025[3]; NeurIPS workshop poster | Agent-based interpretable report metric | Reference and candidate reports | No | Question-level clinical precision and recall proxies |
| Disease-aware dual-stage, 2026[4]; AAAI | Disease tokens, visual fusion, retrieval | CheXpert Plus; IU X-Ray; MIMIC-CXR | No symmetric source-target reversal reported | Aggregate language and clinical metrics |
| TKSG, 2025[5]; ICME | Topic-keyword semantic guidance and retrieval | IU X-Ray; MIMIC-CXR | No symmetric source-target reversal reported | Aggregate generation and clinical metrics |
| Sun et al, 2025[6]; Radiology | LLM detection of report errors | Synthetic and MIMIC-CXR-derived report errors | Not image-to-report transfer | Negation, laterality, interval, transcription errors |
| MedGemma 1.5, 2026[7]; official model card | Open medical multimodal foundation model | Medical image and text tasks; CXR report generation evaluation | No | Aggregate model/task benchmarks |
| Nimalsiri et al, 2023[8]; ICARC | Segmentation-enhanced transformer report generation (MERGIS) | MIMIC-CXR; auxiliary lung-segmentation datasets | No | Language-overlap and generation metrics |
| Edirisinghe et al, 2025[9]; IEEE Access | Abnormality-guided expert-token vision-language model (META-CXR) | MIMIC-CXR and IU X-Ray in model development/evaluation | No symmetric source-target reversal | Abnormality classification and aggregate report metrics |
| Present study | Fixed-system risk audit with an exploratory modern vision-language model stress test | MIMIC-CXR↔IU X-Ray fixed-system transfer | Yes | Validity, recurrence, concentration, prefix, per-label proxy, text audit, human endpoints |

Symmetric bidirectional transfer requires explicit source-target reversal; case-linked provenance traces an endpoint to final text and intermediate labels or scores. Included sources were published or accepted, plus the official model card.

Table S33. Recent prior work: statistical uncertainty, human review, case-linked provenance, and remaining distinction^a^

| **Study / status** | **Statistical uncertainty** | **Human / expert review** | **Case-linked provenance** | **Relevance to this study** |
| --- | --- | --- | --- | --- |
|  |  |  |  |  |
| Delbrouck et al, 2025[1]; ACL | Benchmark analyses rather than transfer-cluster inference | 5 board-certified radiologists | No generator-cell-to-labeler trace | Structured reporting rather than bidirectional domain audit |
| RadEval, 2025[2]; EMNLP demo | Includes statistical testing tools | Expert dataset with >450 significant error labels | Auditable metric inputs; no fixed transfer-cell investigation | Evaluation toolkit rather than a single-system audit |
| ICARE, 2025[3]; NeurIPS workshop poster | Perturbation and reproducibility analyses | Clinician alignment study | Question-answer rationales, without a decoding-to-official-label pipeline | Interpretable metric rather than bidirectional transfer |
| Disease-aware dual-stage, 2026[4]; AAAI | Benchmark comparisons / ablations | Primarily automated | No final-text-to-labeler anomaly trace | Model-development study across datasets |
| TKSG, 2025[5]; ICME | Benchmark comparisons | Primarily automated | No case-linked anomalous-cell audit | Multi-dataset model evaluation |
| Sun et al, 2025[6]; Radiology | Comparative model evaluation | Radiologist review of selected outputs | Text-level task by design | Examines error detection rather than report generation |
| MedGemma 1.5, 2026[7]; official model card | Model evaluation rather than transfer-cluster inference | No matched reader study was reported for this stress-test setting | No bidirectional generator-cell provenance audit was reported | Used here as a frozen exploratory cross-architecture stress test |
| Nimalsiri et al, 2023[8]; ICARC | Benchmark comparisons without transfer-cluster inference | No dedicated clinical reader study was reported | No case-linked label/text anomaly trace | Architecture development rather than cross-domain failure audit |
| Edirisinghe et al, 2025[9]; IEEE Access | Benchmark and ablation analyses | Primarily automated evaluation | No case-linked anomalous-cell audit | Modern model-development study rather than a symmetric transfer audit |
| Present study | Paired cluster bootstrap with direction-specific units | Independent blinded review; consensus and adjudication | Final prediction→labeler raw state→reported cell trace | Primary bidirectional audit with a limited external architectural stress test; no claim of fair model superiority |

Definitions and inclusion criteria follow Table S32.

## Exploratory MedGemma stress-test supplement

### Appendix Text S9. MedGemma 1.5 design, automated results, and quality checks

MedGemma 1.5 4B (revision 91850547d9f0b2fdd21aa7c5f4f3d1a8a52c243b) was evaluated under one deterministic configuration on 575 IU and 401 MIMIC-CXR records, plus a 553-entry frontal-only IU subset. It used no task-specific fine-tuning, matched source training, prompt search, or human review; surface validity was prompt conditioned.

The fixed system message was “You are an expert radiologist.” The user prompt requested a concise findings-and-impression chest-radiograph report without model self-reference. Images were processed as RGB at 896×896 pixels; Table S37 lists the effective parameters.

Generation used transformers 4.57.6, PyTorch 2.6.0+cu118, CUDA 11.8, driver 535.230.02, and 2 RTX 3090 devices at batch size 1. MIMIC-CXR remained local. Float32 replaced unstable float16 generation. Manifest and output SHA-256 hashes were 32f43c369b1023dcab1601818d55aa9027264d47f06a51a79959a500f5261042 and 7ad7045bcd45300891f3fc48829a28843a1fcd34ee07eedfc666abcbb9ec3b35. Standalone clustered intervals used the same audit definitions and remained outside the Holm family.

Table S34. MedGemma 1.5 exploratory automated results^a^

| **Metric** | **IU (N=575)** | **IU frontal-only (N=553)** | **MIMIC-CXR (N=401)** |
| --- | --- | --- | --- |
|  |  |  |  |
| Surface valid rate | 0.9948 | 0.9946 | 0.9776 |
| Exact-duplicate rate | 0.6626 | 0.6637 | 0.1446 |
| Top-5 output share | 0.4417 | 0.4467 | 0.0873 |
| Distinct-1 | 0.0140 | 0.0142 | 0.0173 |
| Distinct-2 | 0.0505 | 0.0504 | 0.0742 |
| Single predefined template-prefix rate | 0.0000 | 0.0000 | 0.0000 |
| Official CheXpert micro-F1 | 0.4722 | 0.4763 | 0.4340 |

The prefix endpoint was 0 because MedGemma generally began with section headings. This did not indicate low concentration: the IU top-5 share was 44.17%.

Table S35. Standalone clustered-bootstrap 95% confidence intervals for MedGemma exploratory endpoints^a^

| **Cohort** | **Endpoint** | **Point estimate** | **95% CI** |
| --- | --- | --- | --- |
|  |  |  |  |
| IU | Surface valid rate | 0.9948 | 0.9878-1.0000 |
| IU | Single predefined template-prefix rate | 0.0000 | 0.0000-0.0000 |
| IU | Official CheXpert micro-F1 | 0.4722 | 0.4353-0.5092 |
| MIMIC-CXR | Surface valid rate | 0.9776 | 0.9630-0.9924 |
| MIMIC-CXR | Single predefined template-prefix rate | 0.0000 | 0.0000-0.0000 |
| MIMIC-CXR | Official CheXpert micro-F1 | 0.4340 | 0.3815-0.4787 |

IU intervals used UID-level clusters, whereas MIMIC-CXR intervals used patient clusters. Both remained outside the primary Holm family.

Table S36. MedGemma generation and text-level quality checks^a^

| **Audit item** | **Finding** | **Interpretation** |
| --- | --- | --- |
|  |  |  |
| Generation completeness | 976/976 nonempty outputs; no duplicate ids | Complete cohort retained |
| 512-token outputs | 4 reports; all visibly looping and truncated; invalid/low information | Retained and flagged as severe tail failures |
| MedGemma prediction encoding characters | 0 MedGemma predictions contained U+FFFD or U+2047 | No MedGemma generated-text encoding failure |
| Reference tokenizer-decoder artifact | 52 image-level MIMIC entries, representing 42 unique studies/decoded reference texts, contained U+2047; 0 contained U+FFFD | Introduced during evaluation reference tokenization/decoding; absent from original reports and final human-audit inputs |
| CheXpert character sensitivity | 0/42 unique texts, 0/52 image entries, and 0 labels changed after U+2047 cleaning | Reference-positive counts and micro-F1 were unchanged; prediction-only Distinct-1/2 were unaffected |
| IU concentration | Top-5 share 44.17% despite 0% single-prefix rate | The narrow prefix endpoint did not capture overall concentration |
| No Finding contribution | Material FP contribution but not the sole driver of micro-F1 | No Finding contributed materially but was not the sole driver of the aggregate score |
| Cardiomegaly FP provenance | Typically explicit “mildly enlarged” or “cardiomegaly” wording | Model-generated positive wording, unlike the primary labeler-trigger anomaly |

Complete per-label and per-sample provenance files were retained. Higher proxy scores were not interpreted as better human-assessed quality because MedGemma did not undergo a reader study.

Table S37. Effective MedGemma generation and reproducibility configuration

| **Field** | **Final value** |
| --- | --- |
|  |  |
| Model / revision | google/medgemma-1.5-4b-it; snapshot and processor revision 91850547d9f0b2fdd21aa7c5f4f3d1a8a52c243b |
| Prompt | System: You are an expert radiologist. User: Generate a concise chest X-ray radiology report for this image. Use radiology report style and include the relevant findings and impression. Do not mention uncertainty about being an AI model. |
| Input processing | RGB; Gemma3Processor/Gemma3ImageProcessor; 896×896; image sequence length 256 |
| Decoding | Greedy; num_beams=1; num_return_sequences=1; do_sample=False |
| Length / penalties | max_new_tokens=512; length_penalty=1.0; repetition_penalty=1.0; no_repeat_ngram_size=0 |
| Stopping / cache | early_stopping=False; min_new_tokens unset; use_cache=True; hybrid cache |
| Token IDs | pad_token_id=0; eos_token_id=[1,106] |
| Inactive inherited fields | temperature=1.0; top_p=1.0; top_k=50; inactive because do_sample=False |
| Precision | float32; float16 smoke test failed with NaN first-token logits and all-padding output |
| Software | transformers 4.57.6; torch 2.6.0+cu118; CUDA runtime 11.8; NVIDIA driver 535.230.02 |
| Hardware / sharding | 2×NVIDIA GeForce RTX 3090; 2 deterministic row-index shards; effective batch size 1 |
| Manifest SHA-256 | 32f43c369b1023dcab1601818d55aa9027264d47f06a51a79959a500f5261042 |
| Merged output SHA-256 | 7ad7045bcd45300891f3fc48829a28843a1fcd34ee07eedfc666abcbb9ec3b35 |

## Operational distinction between the lexical prefix endpoint and human ratings

### Appendix Text S10. Illustrative MIMIC-to-IU cases and interpretive boundary

Tables S38 and S39 show 5 preconsensus MIMIC→IU cases in which exact-prefix status and whole-report genericness diverged. The targeted-panel case was illustrative and excluded from primary summaries. Table S40 separately reconciles complete-report recurrence with consensus informativeness in the 35-case MIMIC→IU ON+B4 primary audit.

Table S38. Illustrative MIMIC-to-IU cases: prediction text and lexical prefix status^a^

| **Case ID** | **Direction** | **Configuration** | **Prediction text** | **Prefix hit** |
| --- | --- | --- | --- | --- |
|  |  |  |  |  |
| TARGETED_FAILURE_PANEL_M2I_016 | MIMIC→IU | ON+G1 | no acute cardiopulmonary process . there is no evidence of pulmonary edema . there are normal . | Yes |
| PRIMARY_REPRESENTATIVE_AUDIT_M2I_041 | MIMIC→IU | ON+G1 | no acute cardiopulmonary process . the right lung volumes are unchanged . | Yes |
| PRIMARY_REPRESENTATIVE_AUDIT_M2I_062 | MIMIC→IU | ON+G1 | no acute cardiopulmonary process . there is no evidence of pulmonary edema . there are normal . | Yes |
| PRIMARY_REPRESENTATIVE_AUDIT_M2I_057 | MIMIC→IU | ON+B4 | in comparison with the study of the right pleural effusion or pneumothorax . | No |
| PRIMARY_REPRESENTATIVE_AUDIT_M2I_064 | MIMIC→IU | ON+K50 | in comparison with the study of the chest provided of the left lung base . no acute osseous structures are stable . | No |

A prefix hit indicates that the normalized text began with “no acute cardiopulmonary process.”

Table S39. Illustrative MIMIC-to-IU cases: original independent reviewer ratings^a^

| **Case ID** | **Template genericness, A/B** | **Major clinically relevant omission, A/B** | **Major unsupported finding, A/B** |
| --- | --- | --- | --- |
|  |  |  |  |
| TARGETED_FAILURE_PANEL_M2I_016 | 0/0 | 0/0 | 0/0 |
| PRIMARY_REPRESENTATIVE_AUDIT_M2I_041 | 1/0 | 0/0 | 1/1 |
| PRIMARY_REPRESENTATIVE_AUDIT_M2I_062 | 1/0 | 1/1 | 0/0 |
| PRIMARY_REPRESENTATIVE_AUDIT_M2I_057 | 2/1 | 1/1 | 1/1 |
| PRIMARY_REPRESENTATIVE_AUDIT_M2I_064 | 1/2 | 1/0 | 1/1 |

A/B denotes the original pre-consensus ratings. Prefix status reflects the opening phrase, whereas genericness reflects the complete report and image. These examples were illustrative rather than inferential.

Table S40. Complete-report recurrence and informativeness in the MIMIC-to-IU ON+B4 primary human-audit subseta

| Category | Full cohort, n | Audit, n | Score 0 | Score 1 | Score 2 | Score 3 | ≥2, n/N |
| --- | --- | --- | --- | --- | --- | --- | --- |
| Top-5 repeated | 562 | 34 | 5 | 12 | 14 | 3 | 17/34 |
| Other repeated | 7 | 1 | 0 | 0 | 0 | 1 | 1/1 |
| Singleton | 6 | 0 | 0 | 0 | 0 | 0 | NA |
| Total | 575 | 35 | 5 | 12 | 14 | 4 | 18/35 |

a Complete-report groups were defined by exact matching of normalized full prediction strings. Repeated indicates a group frequency of at least 2 in the full 575-case cohort. Scores ≥2 met the prespecified informativeness threshold. Recurrent texts could be partially applicable to some cases; this table does not establish individualized generation.

## References

1. Delbrouck JB, Xu J, Moll J, Thomas A, Chen Z, Ostmeier S, et al. Automated structured radiology report generation. In: Proceedings of the 63rd Annual Meeting of the Association for Computational Linguistics (Volume 1: Long Papers); 2025 Jul 27-Aug 1; Vienna, Austria. Stroudsburg, PA: Association for Computational Linguistics; 2025:26813-26829. doi:10.18653/v1/2025.acl-long.1301.

2. Xu J, Zhang X, Abderezaei J, Bauml J, Boodoo R, Haghighi F, et al. RadEval: a framework for radiology text evaluation. In: Proceedings of the 2025 Conference on Empirical Methods in Natural Language Processing: System Demonstrations; 2025 Nov 4-9; Suzhou, China. Stroudsburg, PA: Association for Computational Linguistics; 2025:546-557. doi:10.18653/v1/2025.emnlp-demos.40.

3. Dua R, Kwon YJ, Dogra S, Freedman D, Ruan D, Nashawaty M, et al. Clinically grounded agent-based report evaluation: an interpretable metric for radiology report generation. Poster presented at: Second Workshop on GenAI for Health: Potential, Trust, and Policy Compliance at NeurIPS 2025; 2025 Dec 6; San Diego, CA. URL: https://openreview.net/forum?id=WjfBWxDT6f [accessed 2026-07-05].

4. Wu P, Dong H, Lin Y, Ding Y, Peng Y. A disease-aware dual-stage framework for chest X-ray report generation. Proc AAAI Conf Artif Intell; 2026;40(40):33953-33961. doi:10.1609/aaai.v40i40.40688.

5. Xiao J, Liu H, Dong R, Liu J, Yu H. Automated radiology report generation based on topic-keyword semantic guidance. In: 2025 IEEE International Conference on Multimedia and Expo (ICME); 2025 Jun 30-Jul 4; Nantes, France. Piscataway, NJ: IEEE; 2025:1-6. doi:10.1109/ICME59968.2025.11209777.

6. Sun C, Teichman K, Zhou Y, Critelli B, Nauheim D, Keir G, et al. Generative large language models trained for detecting errors in radiology reports. Radiology; 2025;315(2):e242575. doi:10.1148/radiol.242575. PMID:40392090.

7. Google. MedGemma 1.5 model card. Health AI Developer Foundations. Updated April 21, 2026. URL: https://developers.google.com/health-ai-developer-foundations/medgemma/model-card [accessed 2026-07-05].

8. Nimalsiri W, Hennayake M, Rathnayake K, Ambegoda TD, Meedeniya D. Automated radiology report generation using transformers. In: 2023 3rd International Conference on Advanced Research in Computing (ICARC); 2023 Feb 23-24; Belihuloya, Sri Lanka. Piscataway, NJ: IEEE; 2023:90-95. doi:10.1109/ICARC57651.2023.10145699.

9. Edirisinghe D, Nimalsiri W, Hennayake M, Meedeniya D, Lim G. Chest X-ray report generation using abnormality guided vision language model. IEEE Access; 2025;13:157651-157673. doi:10.1109/ACCESS.2025.3606961.
